# Trends in insulin resistance: insights into mechanisms and therapeutic strategy

**DOI:** 10.1038/s41392-022-01073-0

**Published:** 2022-07-06

**Authors:** Mengwei Li, Xiaowei Chi, Ying Wang, Sarra Setrerrahmane, Wenwei Xie, Hanmei Xu

**Affiliations:** 1 The Engineering Research Center of Synthetic Peptide Drug Discovery and Evaluation of Jiangsu Province, China Pharmaceutical University, Nanjing, 210009 China; 2 State Key Laboratory of Natural Medicines, Ministry of Education, China Pharmaceutical University, Nanjing, 210009 China; 3 Development Center for Medical Science & Technology National Health Commission of the People’s Republic of China, 100044 Beijing, China; 4 NANJING ANJI BIOTECHNOLOGY CO. LTD, Nanjing, 210033 China

**Keywords:** Molecular biology, Pathogenesis

## Abstract

The centenary of insulin discovery represents an important opportunity to transform diabetes from a fatal diagnosis into a medically manageable chronic condition. Insulin is a key peptide hormone and mediates the systemic glucose metabolism in different tissues. Insulin resistance (IR) is a disordered biological response for insulin stimulation through the disruption of different molecular pathways in target tissues. Acquired conditions and genetic factors have been implicated in IR. Recent genetic and biochemical studies suggest that the dysregulated metabolic mediators released by adipose tissue including adipokines, cytokines, chemokines, excess lipids and toxic lipid metabolites promote IR in other tissues. IR is associated with several groups of abnormal syndromes that include obesity, diabetes, metabolic dysfunction-associated fatty liver disease (MAFLD), cardiovascular disease, polycystic ovary syndrome (PCOS), and other abnormalities. Although no medication is specifically approved to treat IR, we summarized the lifestyle changes and pharmacological medications that have been used as efficient intervention to improve insulin sensitivity. Ultimately, the systematic discussion of complex mechanism will help to identify potential new targets and treat the closely associated metabolic syndrome of IR.

## Introduction

**Fig. 1:**
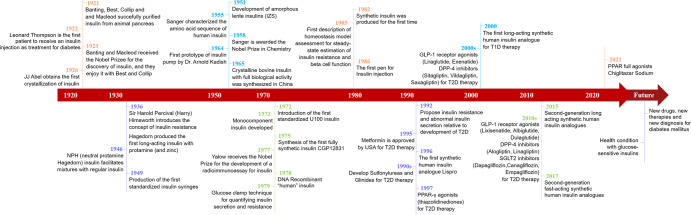
A timeline of key discoveries in our understanding of insulin and insulin resistance

The discovery of insulin in 1921 was a milestone event^1^ that introduced the possibility of systematic research of insulin action (Fig. 1). Frederick Sanger (1918-2013) sequenced bovine insulin in 1955, identifying its exact amino-acids composition,^2,3^ and was awarded with the Nobel Prize for Chemistry in 1958. In 1965, a large team in the People’s Republic of China successfully synthesized the crystalline bovine insulin with full biological activity, immunogenicity and chemical property for the first time in the world.^4^ Subsequently, human insulin was produced using recombinant DNA methods and genetic modification of bacteria.^5^ Insulin therapy and understanding its mechanisms of action become important later research targets. Insulin is a peptide hormone that is produced and released by islets pancreatic β cells, that finely regulates the glucose uptake from blood into liver, fat, and skeletal muscle cells.^6^ Insulin also promotes several other cellular processes, in addition to glucose homeostasis, including regulation of glycogen synthesis, lipid metabolism, DNA synthesis, gene transcription, amino acid transport, protein synthesis and degradation^7^.

Under normal physiological conditions, increased plasma glucose levels lead to increased insulin secretion and circulating insulin levels, thereby stimulating glucose transfer into peripheral tissues and inhibiting hepatic gluconeogenesis. Individuals with defected insulin-stimulated glucose uptake into muscle and adipocytes tissues, in addition to impaired insulin suppression of hepatic glucose output, are described as having ‘insulin resistance’(IR).^8^ Several diseases are clinically associated with IR includes obesity, type 2 diabetes mellitus (T2DM), metabolic syndrome, cardiovascular disease, MAFLD, PCOS, and cancer.^9–13^ Thus, there is an urgent need to identify the mechanisms of IR and effective interventions for treating these metabolic diseases. A relatively safe and well accepted approach in the prevention and treatment of IR is via lifestyle interventions. Nutritional intervention is an important first step that emphasizes a low-calorie and low-fat diet that stimulates excessive insulin demands. In addition, increased physical activity is recommended to help increase energy expenditures and improve muscle insulin sensitivity, this two approach represent the fundamental treatment for IR.^14,15^ The second step is the use of pharmacologic medications, including metformin, oral sulphonylureas, oral sodium-glucose cotransporter 2 (SGLT2) inhibitors, oral dipeptidyl peptidase 4 (DPP-4) inhibitors, oral α-Glucosidase, injectable glucagon-like peptide 1 (GLP1) receptor agonists, or injectable insulin.^16,17^

In this review, the mechanism of insulin action and IR are first described to promote the development of new therapeutic strategies. Further, the direct and indirect effects of insulin on target tissues are discussed to better understand the pivotal role of tissue crosstalk in systemic insulin action. Lastly, diseases associated with IR are discussed and summarized. Many methods and multiple surrogate markers have been developed to calculate the IR. We then summarize the current measurements and potential biomarkers of IR to facilitate the clinical diagnosis. Finally, we provide the general approaches including lifestyle intervention, specific pharmacologic interventions and clinical trials to reduce IR.

## The Insulin signaling and IR

**Fig. 2:**
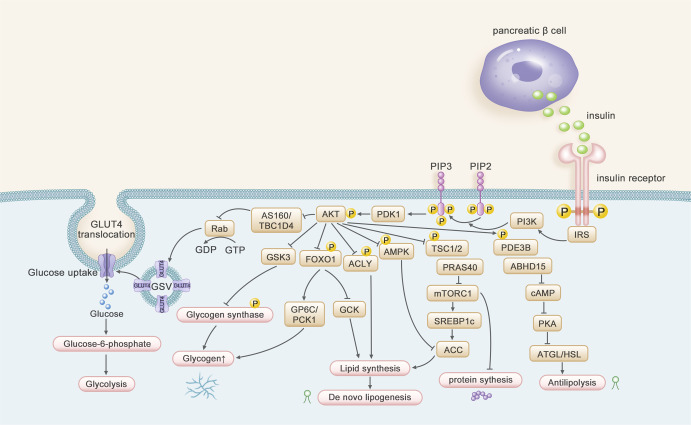
The insulin signaling pathway including proximal and distal segments

Insulin is an endocrine peptide hormone with 51 amino acids and composed of an α and a β chain linked together as a dimer by two disulfide bridges^18^ along with a third intrachain disulfide bridge in the α chain.^19,20^ Insulin is released by pancreatic beta cells and is essential for glucose and lipid homeostasis.^21^ Insulin binds the insulin receptor (INSR) on the plasma membrane of target cells, leading to the recruitment/phosphorylation of downstream proteins, that primarily including insulin receptor substrate (IRS), PI3-kinase(PI3K), and AKT isoforms, that are largely conserved among insulin target tissues and that initiate the insulin response.^22,23^ The pathway diversification of insulin signaling downstream targets of Akt activation leads to different distal signaling in target tissues response to insulin (Fig. 2). (1) AKT substrates include the inactive glycogen synthase kinase 3 (GSK3) and active protein phosphatase 1 (PP1) that promote glycogen synthesis.^24–26^ In addition, the transcription factor forkhead box O1 (FOXO1) is phosphorylated by AKT and is transported from the nucleus, thereby disabling its transcription factor activity and inhibiting gluconeogenesis.^27,28^ (2) Tuberous sclerosis complex 1/2 (TSC1/2) and proline-rich Akt substrate 40 (PRAS40) are inhibitors of mTORC1, thereby inducing protein synthesis and adipogenesis.^29,30^ (3) The upregulation of sterol regulatory element binding protein 1c (SREBP-1c), de-phosphorylation of ACC1/2 through inhibition of AMP-dependent protein kinase (AMPK), and the phosphorylation of ATP citrate lyase (ACLY) lead to constitutive increases in *de novo* lipogenesis (DNL);^31–33^ (4) Phosphodiesterase 3B (PDE3B) and the abhydrolase domain containing 15 (ABHD15) protein are involved in suppression of lipolysis in adipocytes by inhibiting adipose triglyceride lipase (ATGL) and hormone-sensitive lipase (HSL).^34,35^

Accumulation of reports have demonstrated that IR is a complex metabolic disorder with integrated pathophysiology. The exact causes of IR has not been fully determined,^36–38^ but ongoing research seeks to better understand how IR develops. Here, we focus on the underlying mechanism of IR including direct defective of insulin signaling, epidemiological factors, interorgan metabolic crosstalk, metabolic mediators, genetic mutation, epigenetic dysregulation, non-coding RNAs, and gut microbiota dysbiosis.

### The direct defective of insulin signaling

As has been mentioned, the proper modulators acting on different steps of the signaling pathway ensure appropriate biological responses to insulin in different tissues. Thus, the diverse defect in signal transduction contributes to IR.

#### Proximal insulin receptor signaling and IR

Insulin exerts its biological effects by binding to its cell-surface receptors, therby activating specific adapter proteins, such us the insulin receptor substrate (IRS) proteins (principally IRS1 and IRS2), Src-homology 2 (SH2) and protein-tyrosine phosphatase 1B (PTP1B), eventually promoting downstream insulin signaling involving glucose homeostasis.^39^ Thus, changes in insulin receptor expression, ligand binding, phosphorylation states, and/or kinase activity accounts for many IR phenotypes.

Most individuals that are obese or diabetic exhibit decreased surface INSR content and INSR kinase (IRK) activity.^40^ Defective IRK activity is also implicated by decreased IRS1 tyrosine phosphorylation which is consistently observed in insulin-resistant skeletal muscles.^41^ In addition, the specific knockout or ablation of *INSR* in livers prevents insulin suppression of hepatic glucose production (HGP),^42,43^ suggesting a direct role for INSR in hepatic IR. Second, decreased expression or serine phosphorylation of IRS proteins^44,45^ can reduce their binding to PI3K, thereby down-regulating PI3K activation and inducing apparent IR. Third, homozygous mice of *IRS1* or *IRS2* gene leading to peripheral IR and diabetes, and impaired insulin secretion through restrained PI3K/AKT signal transduction.^46^ Thus, pharmacological inhibitors, blocking antibodies and knockdown of PI3K abolishes the insulin stimulation of glucose transport, GLUT4 translocation and DNA synthesis.^47–49^ Additionally, deletion of *Pik3r1* and *Pik3r2* that encode PI3K subunit isoforms in skeletal muscle inhibits insulin-stimulated glucose transport.^50^ Similarly, interfering Akt mutant suppresses insulin-stimulated GLUT4 translocation,^51^ and inhibition of AKT expression, or impairment in AKT Ser473 phosphorylation are certainly detected in both muscle and liver IR.^52,53^ Further, there are three known isoforms of Akt1/2/3 in insulin sensitive tissues, the present study showed that the Akt2 and Akt3 defects impaired insulin-stimulated glucose transport in IR.^54^ In addition, elevated plasma nonesterified fatty acid (NEFA) levels impaired the insulin-induced increase in IRS-1-associated PI3K activity, but no defect in Akt phosphorylation was observed.^55^ Together, the combined actions of various disorders in the proximal signaling components leads to impaired glucose metabolism and IR, and a major challenge remains for understanding IR mechanisms regarding how to distinguish the causes from insulin effects or primary defects from their consequences.

#### Distal downstream signaling and IR

It is generally accepted that diverse downstream targets of Akt activation lead to different distal signaling in target tissues response to insulin. As mentioned above, there are more than 100 Akt substrates mainly including GLUT4, FOXO1, GSK3, mTORC1, SREBP-1c, TSC1/2, PRAS40, ABHD15, PDE3B.^56^ Among them, GLUT4 is the best characterized and mediates glucose uptake in skeletal muscle and white adipose cells after insulin stimulation.^57,58^ Impaired translocation of intracellular GSV (GLUT4 storage vesicles) caused decreased insulin-stimulated glucose uptake which are associated with IR in muscle and adipose tissues.^59^ This proved that heterozygous deletion of Glut4 mice reduce glucose uptake and develop metabolic disease in adipocytes.^60^ Similarly, defection of insulin-stimulated GLUT4 translocation to the cell surface occurs in skeletal muscle in various IR mice models^61,62^ and humans with T2DM.^63,64^ In addition, loss of *Tbc1d4* in mice that phosphorylated by Akt leads to the attenuation of downstream target activation of Rab-GTPase proteins associated with GLUT4 vesicles, and completely abolishes insulin-stimulated adipocyte glucose uptake.^65^ Mice homozygous for the physiologically important AKT substrate TBC1D4 Thr^649^ knock-in exhibit impaired insulin-stimulated myocellular GLUT4 translocation and induction of glucose intolerance.^66^ In summary, continued discovery of novel AKT substrates involved in GLUT4 translocation indicates that many but not all of the same effectors are involved in the glucose uptake of different tissues, and further studies should be conducted to identify the molecular mediators in all phases of insulin-stimulated glucose uptake.

### Epidemiology studies of IR

#### Sex difference

Different investigations have indicated that premenopausal women exhibit many less metabolic disorders than men, including lower incidence of IR, although this effect diminishes severely when women reach the postmenopausal situation.^67,68^ Specifically, female sex hormones including estradiol (e.g., 17β-oestradiol)^69^ protect female proopiomelanocortin (POMC) neurons from IR by enhancing POMC neuronal excitability and coupling insulin receptors to transient receptor potential (TRPC) channel activation. Concomitantly, clinical and experimental observations^70,71^ have revealed that endogenous estrogens can protect against IR primarily through ER-α activation in multiple tissues, including in the brain, liver, skeletal muscle, and adipose tissue, in addition to pancreatic β cells. Further, female hormone estrogens are determinants that mediate body adiposity levels and body fat distribution in addition to glucose metabolism and insulin sensitivity. Specifically, insulin sensitivity and capacities for insulin responses in women is significantly higher than men.^72^ Women younger than 51 had a significantly lower fasting glucose and triglyceride concentration compared with men.^73^ Furthermore, sex differences are associated with genetic polymorphisms in the development of IR and diabetes. Male homozygous for the polymorphism of *PPP1R3A* gene that involved in glycogen synthase activity are significantly younger at diagnosis than female.^74^ The difference in regard to visceral, hepatic adiposity, hypoadiponectinemia, the insulin-sensitizing hormone-adiponectin, resting energy expenditure,^67,75,76^ lipid metabolism^77^ may also contribute to higher IR in male compared with female. Thus, additional studies are required to understand mechanisms underlying sex differences and IR development.

#### Ethnicity difference

T2DM is anticipated to impact nearly 600 million people globally by 2035,^78^ much of the investigations have recognized that the prevalence of T2DM are affected by different race/ethnicity, partly because of the differences in insulin sensitivity which affects plasma triglyceride levels.^79–81^ For example, Singapore Adults Metabolism Study (SAMS) performed a sub-group analysis and observed that Chinese and Malays exhibit greater insulin-sensitivity compared with Asian Indians among lean and young Singaporean males,^82^ the previous reports was further confirmed that the prevalence of T2DM in Chinese (9.7%) and Malays (16.6%) are lower than Asian Indians (17.2%).^83^ In the United Kingdom. South Asian children exhibit greater IR compared with white European children, while girls are more insulin resistant than boys, with sex and ethnicity differences related to insulin sensitivity and body composition.^84,85^ In addition, individuals of Aboriginal or South Asian descent (among Aboriginal, Chinese, and Indian populations) that also exhibit increased levels of body fat and visceral fat deposition appear to have a greater propensity for IR and type 2 diabetes.^86^ These interesting findings force us to reconsider the effect of the ethnic differences in IR, which is important to reduce the morbidity and mortality related to diabetes mellitus and metabolic syndrome.

#### Modifiable lifestyle factors

Despite the above objective factors, some modifiable lifestyle factors including diet, exercise, smoking, sleep and stress are also considered to contribute to IR.^87–89^ For instance, irregular daily eating habits or poor sleep are connected to elevated risk for both obesity and IR. Further, circadian clocks disruption might also be an important factor to IR development via various factors including clock gene mutations, disturbed sleep cycles, shift work and jet lag.^90,91^ Moreover, epidemiologic studies of different institutions showed that individuals with regular exercise, healthy diet (including more soluble fiber, colorful fruit, vegetables, green tea, or less intake of added sugars, carbs, trans-fats), limiting alcohol intake, avoiding smoking cigarettes and reduced levels of stress, which indeed increase insulin sensitivity.^92–94^ Collectively, there are many relatively simple things we can do to naturally increase insulin sensitivity but ensure professional healthcare consultant first before adding medication regimen.

Different investigations suggest that vitamin D supplementation might reduce IR in some people due to increasing insulin receptor genes transcription and anti-inflammatory properties,^95^ while some researchers found that Vitamin D has no effect on IR.^96^ Thus, further studies should be performed to discover more about the mechanism and the effect of vitamin D on insulin resistance. Both experimental (animals) and clinical studies have shown that many hormones can induce IR including glucocorticoids (GCs),^97^ cortisol,^98^ growth hormone,^99^ and human placental lactogen,^100^ which may decrease the insulin-suppressive effects on glucose production and reduce the insulin-stimulated glucose uptake. Several other clinical medications including anti-adrenergic (such as salbutamol, salmeterol, and formoterol),^101^ HIV protease inhibitors,^102,103^ atypical antipsychotics^104^ and some exogenous insulin^105^ that may improve IR because of the disordered insulin signaling. All together, there may have synergistic effects of different risk factors on insulin resistance, scientific researchers should cooperate with medical experts to reduce the chances of becoming insulin resistant.

### The interorgan metabolic crosstalk in IR

**Fig. 3:**
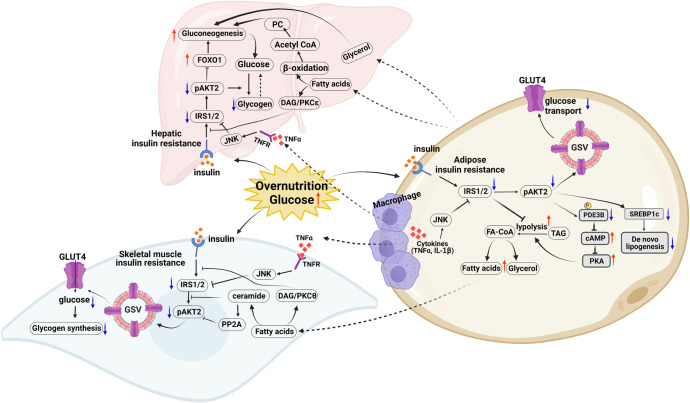
An integrated physiological signaling on different target tissues insulin resistance

As discribed above, insulin signaling calibrates glucose homeostasis by limiting hepatic glucose output via decreased gluconeogenesis and glycogenolysis activities. These processes consequently increase the glucose uptake rates in muscle and adipose tissues. In addition, insulin profoundly affects lipid metabolism by increasing lipid synthesis in liver and fat cells (Fig. 3), in addition to switching-off fatty acid release from triglycerides (TG) in fat and muscle tissues.^106^

Despite stimulated glucose uptake, insulin rapidly reduces hepatic glucose output and hepatic glucose production (HGP) by activating glycogen synthesis, and suppressing glycogenolysis and gluconeogenesis in liver.^107,108^ Further, gluconeogenesis suppression by insulin is mediated by inhibition of FOXO1 transcription factors.^109^ For example, some mouse models of profound hepatic IR exhibit increased *G6pc* (glucose-6-phosphatase) expression suggesting increased FOXO1 activity.^110^ Moreover, correct hepatocellular insulin action also carries suppression of hepatic glycogenolysis and stimulation of glycogenesis.^111^ A remaining question is whether potential controlling factors including allosteric control of glycogen synthase (GS) and phosphorylases via glucose-6-phosphate (G6P),^112^ in addition to the insulin-independent transport of glucose across the hepatocellular plasma membrane by GLUT2 (as a glucose-sensitive protein in liver cells), can impact hepatic glycogen metabolism.^113,114^ Loss of GLUT2 leads to a typical combination of hepatic glycogen accumulation, glucose intolerance, and fasting hypoglycemia.^115^ In addition, liver insulin also effectively regulates lipid metabolism primarily by promoting cleavage and nuclear translocation of the sterol regulatory element binding protein 1c (SREBP-1c)^116,117^ that activates lipogenesis in hepatocytes. Insulin induces SREBP-1c maturation via a proteolytic mechanism started in the endoplasmic reticulum (ER), wherein hepatic IR is highly associated with hepatic steatosis.^118^ Over-expression of liver SREBP-1c has been described in several IR models of including IRS2 knockout,^119^ lipodystrophic and ob/ob mice.^120^ In addition, hepatic glucose lead to a deficiency in the transcription factor carbohydrate responsive element binding protein (ChREBP) resulting in reduced mRNA levels of glycolytic and lipogenic enzymes, as well as SREBP-1c levels. Accordingly, restoration of nuclear SREBP-1c expression in liver-specific *Chrebp* defective mice normalized expression of some lipogenic genes, while not affecting glycolytic genes expressing. In contrast, ChREBP overexpression alone failed to promote the expression of lipogenic genes in the livers of mice lacking active SREBPs. Together, these data demonstrate that SREBP-1c mediates the induction of insulin lipogenic genes, but that SREBP-1c and ChREBP are both necessary for harmonious induction of glycolytic and lipogenic genes.^121^ ChREBP inhibition markedly decreased the expression of L-PK, ACC, and FAS, but restored liver insulin sensitivity by restoring protein kinase B (Akt), and Foxo1 phosphorylation activity with insulin.^122^ This paradoxical response of SREBP-1c and ChREBP in hepatic IR is probably due to many branch effectors (including PI3K/AKT, AMPK, mTORC1, and FOXO1) involved in the hepatic glucose and lipid metabolism. Altogether, these above pathways and components can be used to clarify the popular pathophysiology of hepatic IR.

The lipid metabolisms including increased *de novo* lipogenesis and attenuation of lipolysis in the adipose tissue largely coordinate with glucose homeostasis response to insulin stimulation. De novo lipogenesis regulation in adipose is similar to that in livers, wherein adipose-ChREBP is a major determinant of adipose tissue fatty acid production and systemic insulin sensitivity, that is induced by GLUT4-mediated glucose uptake, and genetically ablating ChREBP impairs insulin sensitivity in adipose tissue^123^ In addition, lipogenic gene *FASN* and *DGAT* mRNA expression in adipose tissue have been shown to correlate strongly and positively with insulin sensitivity, which were may reduced by larger adipocytes in adipose tissue of obese individuals. The lipogenesis stimulation of insulin is also reduced in larger, more insulin-resistant cells. Insulin suppression of lipolysis includes the hydrolytic cleavage of triglycerides, resulting in the generation of fatty acids and glycerol. The best understood effectors for this process are PDE3B and ABHD15 that operated by the suppression of cAMP to attenuate pro-lipolytic PKA signaling toward adipose triglyceride lipase (ATGL), hormone-sensitive lipase (HSL), and perilipin (PLIN).^34,124,125^ Phosphorylation and activation of PDE3B at Ser^273^and Ser^296^ by AKT is a key event in antilipolysis after insulin stimulation. Further, inhibition of PDE3B inhibits insulin-induced glucose uptake and antilipolysis.^126^ *Pde3b* knockout mice exhibit impaired suppression of plasma NEFA levels and corresponding impairment in hepatic glucose production suppression during glucose tolerance tests.^127^ Further, mice homozygous for the *Plin1* deletion exhibit high basal lipolysis and are unresponsive to adrenergic stimulation, confirming that PLIN is actively controls lipolysis^128,129^ Taken together, all the evidence supports that white adipose tissue (WAT) lipolysis suppression of insulin also associated with hepatic gluconeogenes, and the mechanisms of IR include complex mediators and metabolic networks.

Insulin stimulated protein synthesis is mediated by activation of the protein kinases Akt and mTOR (specifically mTORC1 and mTORC2) in numerous insulin-responsive cell types, such as hepatocytes, adipocytes, and myocytes.^130^ AKT also phosphorylates the TSC1-TSC2 complex to relieve its inhibition on the mTORC1. Inhibition of mTOR by rapamycin obviously impairs insulin-activated protein synthesis.^131^ Recent studies have identified that Akt phosphorylates and inactivates the PRAS40 (proline-rich Akt/PKB substrate 40), which abates its binding inhibition of mTOR signaling and promotes protein synthesis. Amino acids metabolic substrates enhance insulin sensitivity and responsiveness of the protein synthesis system by increasing mTOR activity and inhibiting protein degradation in liver, muscle, and heart tissues.^132,133^ Skeletal muscle tissue is the largest protein/amino acid (AA) reservoir in the human body, and lower muscle protein synthesis (MPS) induces fed-state anabolic resistance,^134^ in addition to exercise training of the Akt/mTOR pathway. These processes, in turn, promote protein synthesis and antagonize protein degradation.^135^ Further, PRAS40 and mTOR also exerts negative feedbacks on proximal insulin signaling, PRAS40 knockdown significantly decreases Akt phosphorylation, mTORC1 binds and inhibits INSR by inducing destabilization of IRS1.^28^ The signaling system of IR are multifactorial including different metabolic pathways, such as glucose, lipid, and protein, identifying new molecules will be crucial to unraveling more effective treatment of IR and associated metabolic diseases.

### The contribution of metabolic mediators to IR

#### Adipokines dysregulation and IR

Adipose tissue can secrete various bioactive circulating mediators referred as ‘adipokines’, like adiponectin, leptin, chemerin, resistin, visfatin and vaspin, in addition to cytokines and chemokines such as tumor necrosis factor-alpha (TNF-α), interleukin-6 (IL-6), IL-1β, and monocyte chemoattractant protein-1.^136^ Dysregulation of these adipokines has been implicated in the onset of obesity, IR, type 2 diabetes, cardiovascular disease, hypertension and metabolic syndromes.^137^

Adiponectin is the most abundant protein secreted by adipose tissue and exhibits potent anti-inflammatory properties.^138^ In contrast to other adipokines, plasma adiponectin levels were reduced by pro-inflammatory factors like TNF-α, IL-6, ROS, and hypoxia in animal models of obesity and IR.^139,140^ Adiponectin activates the AMPK and PPAR-α signaling pathways through adiponectin receptor 1 (AdipoR1) and AdipoR2 respectively, leading to enhanced fatty acid oxidation and glucose uptake in muscle, along with suppressed gluconeogenesis in liver tissues. Moreover, targeted disruption of AdipoR1 results in halted adiponectin-induced AMPK activation, increased endogenous glucose production and increased IR. Similarly, AdipoR2 deletion results in decreased PPAR-α signaling pathway activity and IR. In addition, chemerin is a chemokine highly expressed in liver and white adipose tissue that regulates the expression of adipocyte genes involved in glucose and lipid homeostasis like IRS-1 tyrosine phosphorylation activity, GLUT4, fatty acid synthase and adiponectin. Thus, chemerin may increase insulin sensitivity in adipose tissue.^141,142^ Adipocytes also produce apelin that is increased in insulin resistant individuals^143^ and morbidly obese subjects with T2DM.^144^ However, apelin enhances glucose uptake and Akt phosphorylation through AMPK pathway to improve glucose homeostasis and insulin sensitivity,^145^ apelin deficient mice are insulin resistant and have decreased skeletal muscle Akt phosphorylation.^146^ The precise role of anti-inflammatory adipokine in regulating IR require further investigation.

Leptin is a cytokine encoded by *ob* gene and produced by the adipocytes.^147^ Leptin binds to the leptin receptor (LepRb) and activates JAK2/STAT3 pathway to decrease body weight and normalizes blood glucose concentration, meanwhile, JAK2 stimulates the phosphorylation of insulin receptor substrate (IRS1/2), then activates PI3K/Akt pathway and directly affect insulin action.^148^ Leptin limits insulin synthesis and secretion from pancreatic β-cells, resulting in increased insulin sensitivity, reduced hepatic glucose production and decreased glucagon levels.^149,150^ In turn, insulin also plays a role in leptin production stimulation and secretion in adipose tissues.^151^ Leptin-deficient mice (ob/ob) causes both obesity and diabetes due to hyperphagia and blunted metabolic rate, and treatment with exogenous leptin could prevent and reverse the obese phenotype.^152^ Moreover, the decreased permeability of the brain-blood barrier (BBB) to leptin and impaired leptin signal transduction in neurons will lead to leptin resistance in obesity.^148,153^ Specifically, high serum leptin levels connected to IR likely promote the the release of pro-inflammatory compounds that include IL-6, TNF-α, and IL-12 by monocytes and macrophages.^154^ Together, leptin and insulin share pivotal roles in the regulating glucose metabolism, and additional studies are needed to understand the effects of leptin on glucose-insulin homeostasis. In summary, adipose tissue is a central node for distinct adipokines and bioactive mediators in IR pathophysiology. Consequently, identifying the effects of new adipokines will help in the development of new therapeutic strategies for obesity-induced diseases.

#### Fatty acid/lipid metabolism in IR

The specific insulin actions in adipose tissue include activation of glucose uptake and triglyceride synthesis, suppression of triglyceride hydrolysis and free fatty acids (FFA) and glycerol release into the blood circulation.^155–157^ Among these actions, an extremely important function of adipose tissue is via the switches that favor lipids storage in adipocytes over their release into circulation upon need. Once the adipose tissue expandability exceeded limit under overnutrition, excess lipids and toxic lipid metabolites (FFA, diacylglycerol, ceramide) accumulated in non-adipose tissues, thus leading to lipid-induced toxicity (lipotoxicity) and developed IR in liver and muscle.^158,159^ The early mechanism of lipid accumulation pouch plasma fatty acid to induce the IR as detailed in glucose-fatty acid cycle proposed by Randle and colleagues.^160,161^ Their hypothesis suggested that available fatty acids promote fatty acid oxidation and cause the accumulation of mitochondrial acetyl coenzyme A (CoA) and NADH, with subsequent inactivation of pyruvate dehydrogenase. This process would in turn induce increased intracellular citrate levels, thereby inhibiting glucose-6-phosphate (G6P) accumulation. Increased G6P levels then result in decreased hexokinase activity, increased glucose accumulation, and reduced glucose uptake.

Other studies have demonstrated the relevance of the glucose-fatty acid cycle to lipid-induced IR. For example, lipid infusions combined with heparin can be used to activate lipoprotein lipase, thereby increasing plasma concentrations of fatty acids. Further, these infusions promote muscle lipid accumulation and effectively induce IR.^162,163^ In addition, contrary to predicted increases based on the Randle hypothesis, elevated free fatty acid levels were associated with reduced intracellular G6P levels in acute lipid-induced IR and type 2 diabetics.^158,164^ In parallel, lipid-induced IR in skeletal muscle leads to defected insulin signaling and decreased insulin-stimulated glucose transport mediated by GLUT4 translocation, and not by glycolysis inhibition as Randle hypothesized.^165^ Together, these researches suggest that lipid ectopic accumulation is directly correlated with IR.

#### Diacylglycerol and ceramide accumulation

Consistent with the above studies, elevated plasma fatty acid concentrations can result in increased intracellular diacylglycerol (DAG) levels, leading to the activation of protein kinase C isoform (PKC-θ) and PKC-ε isoforms in skeletal muscles and liver respectively. These processes, in turn, decrease insulin-stimulated IRS-1/IRS-2 tyrosine phosphorylation, PI3K activation and downstream insulin signaling that then induces IR in muscles and livers.^166–168^ Deletion of PKCθ in mice inhibits muscle IR is induced by lipid infusion.^169^ Moreover, knockdown of PKC-ε in rats leads to protection from fat-induced hepatic IR,^170^ these results confirm the distinct roles of PKC-θ and PKC-ε in fat-induced IR in skeletal muscles and livers, respectively. Since diacylglycerol acyltransferase 1 (DGAT1) can increase the conversion of DAG into triacylglycerol (TAG),^171^ DGAT1 overexpression could decrease DAG levels and improve insulin sensitivity partially attenuating the fat-induced activation of DAG-responsive PKCs.^172,173^ Conversely, DGAT1 ablation may result in elevated DAG levels and lipid-induced IR. Taken together, these studies strongly support that DAG as a key intermediate of TAG synthesis from fatty acids has central modulation and potential therapeutic values in IR.

Ceramide is another specific lipid metabolite that increases in concentration, along with DAG, in association with IR in obese mice.^174^ Accumulated ceramide mediates the activation of protein phosphatase 2 A (PP2A) and impairs insulin-dependent activation, in addition to signaling of PI3K/Akt by PKCζ,^175^ thereby also disrupting lipid metabolism by inhibiting oxidation and stimulating fatty acid uptake.^176^ In particular, C18- and C16-long-chain ceramides that are produced by ceramide synthase isoforms (CerS1, CerS5, and CerS6) have higher concentrations in insulin-resistant tissues.^177,178^ Consistently, C18-derived ceramides play an important role in fat-induced skeletal muscle IR.^179^ Likewise, C16-ceramides exist in higher concentrations in obese adipose tissue and are associated with hepatic IR.^180,181^ Several lines of evidence suggest that circulating adiponectin is closely related to ceramide concentrations, wherein adiponectin increases ceramidase activity associated with its two receptors AdipoR1/AdipoR2, while lower concentrations of circulating adiponectin increases ceramide concentrations in different tissues.^182,183^ Furthermore, inhibited ceramide synthesis or stimulation of ceramide degradation^176^ can prevent the effects of ceramide on Akt/PKB activation and improve insulin signaling. Thus, ectopic lipid metabolite concentrations (e.g., diacylglycerols and ceramides) may be mechanistic factors underlying liver and muscle IR. Consequently, concerted efforts to decrease lipid components in these organs are the most efficacious therapeutic targets for treating IR and metabolic diseases.

### Genetic mutations in IR

Some human genetic studies indicated that different genomic loci were associated with fasting insulin levels, higher triglyceride and lower HDL cholesterol levels,^184,185^ which are different hallmarks of IR.^186^ Epidemiological and family genetic studies have provided considerable evidence for the genetic basis of both IR and the individual components of the metabolic syndromes.^187,188^ Since 2007, genome-wide association studies (GWAS) and next-generation sequencing (NGS) have identified different genetic variants associated with IR, including *PPARγ*, *IRS1*, *IGF1*, *NAT2*, *KLF14*, *GCKR*, *FTO*, *TCF7L2*, *TMEM163*, *MC4R*, *SC4MOL*, *TCERG1L*, and *ARL15*^184,189,190^ by influencing insulin action via different regulatory mechanisms.

The peroxisome proliferator-activated receptor gamma (*PPARγ*) variant Pro12Ala was one of the first genetic variants identified that is involved in fatty acid and energy metabolism and that is associated with a low risk of developing T2DM.^191^ Variants (A allele to G allele) within IGF-1 (insulin-like growth factor 1) contribute to its low plasma levels, and cause a reduction in insulin sensitivity.^192^ A variant in N-acetyltransferase 2 (NAT2) was recently identified as an insulin sensitivity gene.^193^ Adiponectin is an adipokine involved in improving insulin sensitivity, variants within ADP ribosylation factor like GTPase 15 (*ARL15*) are associated with decreased adiponectin levels and nominally associated with IR.^194^

Despite these potential genetic correlates, variants account for only 25% to 44% of the heritability of IR.^195^ Consequently, the contributions of low-frequency and rare genetic variants towards the heritability of IR have also been explored through a combination of both genome and exosome sequencing.^196,197^ Such studies have identified a low-frequency variant of *CD300LG* that is associated with fasting high-density lipoprotein cholesterol (HDL-C),^198^ and a *TBC1D4* variant that together are connected to higher fasting glucose levels and reduced insulin sensitivity.^199^ The rapid development of genomics methods has enabled considerable progress towards the identification of genetic loci associated with IR by direct or indirect effects. Nevertheless, additional studies are needed to assess the functional relationships between the genetic variants and IR, that are also influenced by various lifestyle and environmental factors.

### Epigenetic dysregulation in IR

Recent studies have suggested that epigenetic modifications such as DNA methylation (DNAm) and histone post-translational modifications (PTM) are implicated in the development of systemic IR.^200,201^

#### DNA methylation

Global and site-specific DNA methylation is generally mediated by DNA methyltransferases (DNMTs). These processes mainly occur in the context of CG dinucleotides (CpGs) and promoter region, while also involving covalent addition or removal of methyl groups as a means to repress or stimulate transcription, respectively.^202^

Firstly, DNA methylation regulates different insulin signaling genes, such as insulin (*INS*), insulin receptor substrate 1 (*IRS1*), Insulin-like growth factor-1/2 (IGF-1/2), Insulin-like growth factor-binding protein 1/2 (IGFBP-1/2), phosphatidylinositol 3-kinase regulatory subunit (PIK3R1), and Glucagon-like peptide-1 receptor (GLP-1R).^203–206^ The methylation status of these genes was found to be altered in obesity and IR. For example, increased *INS* promoter methylation levels and *INS* mRNA suppression were observed under over-nutrition conditions and obese T2DM patients.^207^ Similarly, a research with blood samples of T2DM found that increased *IGF-1* DNA methylation were associated with reduced IGF-I serum levels in T2DM patients.^208^ Insulin-like growth factor binding proteins 1 and 2 (IGFBP1 and IGFBP2, respectively) are the most abundant circulating IGFBPs and have lower concentrations in adipose tissue in obese patients, in addition to being negatively associated with hyperinsulinemia and IR.^209^ Several studies have indicated that increased *IGFBP-1* DNA methylation levels and decreased IGFBP-1 serum levels are associated with newly diagnosed T2DM. Another study demonstrated^210^ that increased *IGFBP2* DNA methylation levels were are associated with lower mRNA expression levels in Visceral Adipose tissue (VAT) of abdominal obesity. Moreover, the first global genome-wide epigenetic analysis in VAT^211^ from IR and insulin-sensitive (IS) morbidly obese patients identified a novel IR-related gene, the zinc finger protein 714 (ZNF714) exhibited the highest DNA methylation difference, and its methylation levels is lower in IR patient than in IS patient, consistent with increased transcription levels, such studies provide potential epigenetic biomarkers related to IR in addition to novel treatment targets for the prevention and treatment of metabolic disorders.

Some DNA methylation in the promoter regions of specific genes related to lipid metabolism (PPARG, PPARA),^212^ low-density lipoprotein receptor-related protein 1 (LRP1),^213^ lipoprotein lipase (LPL),^214^ SREBF1/2,^215^ and inflammation (stearoyl-CoA desaturase 1 (SCD1), chemokine C-C motif chemokine ligand 2 (CCL2), TNF-α, and TGF-β1)^216,217^ are associated with adipose tissue dysfunction and could lead to metabolic disorders. For example, peroxisome proliferator-activated receptor-α and -γ (PPAR-α and PPAR-γ, respectively) are encoded by *PPARA* and *PPARG*, respectively, and they are the two primary nuclear peroxisome proliferator-activated receptors involved in lipid metabolism. Higher *PPARA and PPARG* methylation levels were observed in association with obesity, consistent with decreased PPAR-α and PPAR-γ protein expression levels,^218^ that lead to dyslipidemia and IR. SLC19A1, a gene encoding a membrane folate carrier, was reduced in obese WAT and induced global DNA hypermethylation of chemokine C-C motif chemokine ligand 2 (CCL2) that is a key factor in WAT inflammation,^219^ resulting in increased CCL2 protein secretion and the development of IR in obese.

In addition, several genes methylation involved in hypoxia stress and endoplasmic reticulum stress were regulated in obesity related metabolic diseases.^220^ Hypoxia-inducible factor-3α (HIF3A) belongs to the hypoxia-inducible factors family (HIFs) that play important roles in the pathogenesis of obesity-induced IR, adipose tissue-inflammation and the etiology of obesity related disease. Recent epigenetic genome-wide analysis identified low *HIF3A* methylation levels upregulates HIF3A expression in adipose tissue, thereby leading to adipose tissue dysfunction and adiposity.^221^ Further, reduced *HIF3A* methylation and increased HIF3A levels in blood are associated with IR and higher body mass index (BMI) values in T2DM patients.^222^ The major function of the endoplasmic reticulum (ER) is the synthesis and folding of secreted and transmembrane proteins, increasing evidence suggests that persistent ER stress is associated with the onset and progress of chronic metabolic disorders like obesity and IR. Ramos-Lopez et al.^220^ found that the methylation levels of four ER genes including *ERO1LB*, *NFE2L2*, *MBTPS1* and *EIF2AK4* which encoded ER oxidoreductin-1β (ERO1β), nuclear factor-erythroid 2-related factor (Nrf2), site-1 protease (S1P) and eIF-2-alpha kinase (GCN2) respectively, were strongly correlated with total body fat levels. Specifically, increased insulin concentrations and HOMA-IR index were accompanied by lower ERO1LB and NFE2L2 methylation levels.^223^ Hence, related to hypoxia and ER stress genes could be considered as precise therapeutics to control the IR.

#### Histone modifications

The histone modification effect on gene expression mainly includes histones methylation and acetylation. Histone methylation could either activate gene transcription (H3K4, H3K36, and H3K79) or silence gene expression (H3K9 and H3K27), which depends on the modification site.^224^ Several studies have reported various histone epigenetic modifications of metabolic and mitogenic cascade-related genes of insulin signaling during IR.^225^ PPARG is a key transcription factors that regulates insulin sensitivity, lower histone H3 acetylation and methylation of the *PPARG* gene are associated with reduced PPARG expression that is associated with IR.^212^ Further, increased expression and low methylation of CDKN1A and PDE7B genes in T2DM can lead to impaired insulin release that is stimulated by glucose in T2DM patients.^226^ The high level of H3K4 trimethyl (H3K4me3) of Fxyd3 gene negatively regulates the glucose capacity of insulin-secreting cells in mice.^227^

Histone acetylation increases the accessibility and gene expression of various transcription factors by reducing the positive charge and histone affinity for DNA.^228^ Histone deacetylation is considered to be the inhibition of DNA assembly by chromatin condensation and transcription factors, resulting in transcriptional inhibition. Increasing evidence indicates^229,230^ that IGFR, InsR, IRS1, Akt, GLUT4, and PPAR are more deacetylated in association with IR than in normal physiological conditions. In contrast, IRS2, FoxO, JNK, and AMPK are usually acetylated in association with IR. Castellano-Castillo, D., et al.^231^ utilized a chromatin immunoprecipitation (ChIP) assay to determine that the human adipose tissue H3K4me3 histone mark site in adipogenic, lipid metabolism, and inflammatory genes (such as *LEP*, *LPL*, *SREBF2*, *SCD1*, *PPARG*, *IL6*, *TNF*, and *E2F1*) is positively associated with BMI and HOMA-IR. Further, global proteomic analyses have revealed 15 histone modifications that are differentially abundant in hepatic IR.^232^ These observations provide evidence for diabetes-related histone modification and related impaired insulin release.

### non-coding RNA regulation in IR

Non-coding RNAs (ncRNAs) comprise approximately 98% of human genome transcripts and are generally not translated into proteins.^233^ The rising studies^234,235^ have shown that ncRNAs include microRNAs (miRNAs), long non-coding RNAs (lncRNAs) and circular RNAs (circRNAs) are key mediators in the pathogenesis and progression of metabolic homeostasis.

#### MiRNAs and IR

MicroRNAs (miRNAs) are small ncRNAs (18-22 nucleotides) incorporated into Argonaute (Ago) protein to form miRISCs, which can inhibit the expression of partially or completely complementary target mRMAs.^236^ Dysregulation of various miRNAs within different fluids (e.g., blood, saliva, and urine) is associated with obesity and IR development, including β-cell dysfunction, glucose and lipid homeostasis, and chronic inflammation.^237^

Firstly, pancreatic β cell mass due to dysfunction and/or death are the major cause of insufficient insulin secretion, and the main common mechanisms of T1DM and T2DM. Several miRNAs are involved in β cell differentiation and mature β cell functioning. For example, islet-specific miR-375 overexpression represses glucose-stimulated insulin secretion (GSIS) and insulin gene transcription, that is then reversed upon miR-375 inhibition.^238^ Further, deregulated plasma levels of miR-375 together with miR-150, miR-30a-5p, and miR-15a are observed before T2DM and pre-diabetes onset. Thus, these markers may improve disease prediction and prevention in individuals at high risk for T2DM.^239^ Other miRNAs have been implicated in β-cell proliferation and insulin secretion regulation during IR including miR-124a2, miR-204, miR-184 and miR-24. Further, miR-124a2 targets the genes encoding cAMP-response-element binding protein (creb-1) and forkhead/winged helix transcription factor boxa2 (foxA2) mRNA.^240^ FoxA2 is an upstream regulator of pancreatic duodenal homeobox 1 (pdx1) that is essential for pancreatic development and glucose homeostasis. Furthermore, pdx1, neurogenin-3 (ngn3), and a transcriptional factor essential for insulin transcription (MafA) are essential transcription factors for β-cell differentiation. Thus, miR-124a2 can directly modulate insulin expression through foxA2 and then pdx1. miR-204 expression is induced by the cellular redox regulator thioredoxin-interacting protein (TXNIP) that then represses MafA, thereby inhibiting insulin production.^241^ In addition, miR-185-5p overexpression enhances insulin secretion and promotes pancreatic β-cell proliferation by targeting SOCS3 and regulating the Stat3 pathway.^242^

Numerous studies suggest that miRNAs have pivotal roles in glucose and lipid metabolism.^243,244^ As we mentioned above, glucose metabolism contains different processes including glucose transport, glucose uptake, gluconeogenesis and glycogenolysis. miR-93 was first reported to directly regulate GLUT4 expression in adipocytes.^245^ Further, miR-29 and miR-31 regulate GLUT4 expression in skeletal muscle and adipose tissues of T2DM patients,^246^ respectively. In addition, miR-27a/b levels are higher in the sera of patients with type 2 diabetes, while miR-27a/b overexpression suppresses hepatic glucose output and alleviates hyperglycemia by targeting FOXO1.^247^ Moreover, elevated miR-338-3p levels are responsible for decreased glycogenolysis and subsequent glycogen accumulation by directly targeting the glycogen phosphorylase brain form (PYGB).^248^ miR-185-5p overexpression in db/db mice that represent genetic diabetes models leads to alleviated blood hyperglycemia and decreased gluconeogenesis by directly targeting glucose-6-phosphatase (G6Pase). In addition, the anti-diabetic drug metformin can up-regulate miR-185-5p expression to suppress G6Pase and inhibit hepatic gluconeogenesis.^249^

The balance of low-density lipoprotein (LDL) and high-density lipoprotein (HDL) molecules that are synthesized in hepatocytes is critical for lipid homeostasis. Many miRNAs have been identified as critical regulators of HDL and LDL biogenesis. For example, miR-33, miR-128-1, miR-144, and miR-148a repress expression of the ATP-binding cassette transporter ABCA1 that mediates hepatic HDL generation.^250–252^ Thus, inhibition of these miRNAs increases circulating HDL levels. In addition, miR-30c targets the gene encoding microsomal triglyceride transfer protein (MTP) that is required for the lipidation of newly synthesized APOB in the liver for LD lipoprotein production. miR-30c overexpression reduces the assembly and secretion of these APOB-containing lipoproteins, resulting in decreased plasma LDL levels.^253^ The LDL receptor (LDLR) of hepatocytes is highly expressed and binds LDLs, clearing them from circulation. miR-148a and miR-128-1 repress LDLR expression and inhibition of these miRNAs results in enhanced LDLR expression and clearance of circulating LDL. Further, miR-224 and miR-520d target the LDLR chaperonin PCSK9 and IDOL in addition to the rate-limiting enzyme in cholesterol biosynthesis, HMGCR.^254^ In addition, inhibition of PCSK9, IDOL, and HMGCR by miR-224 and miR-520d was associated with increased LDLR protein levels and increased LDL binding, resulting in decreased plasma LDL cholesterol levels.

Chronic inflammation in insulin-reactive tissues is one of the most important causes of IR and increasing evidence suggests that miRNAs has a pivotal role in the inflammatory process. Obesity inhibited miR-30 expression in adipose tissue macrophages (ATMs), and miR-30 was shown to target Delta-like-4 (DLL4), a Notch1 ligand is associated with ATM inflammation.^255^ miR-30 inhibition triggers Notch1 signaling, pro-inflammatory cytokine (TNFα and CCL2) production, and M1 macrophage polarization, indicating that miR-30 manipulation could be a therapeutic approach for reducing obesity-induced inflammation. Conversely, Wang et al. discovered that miR-1249-3p is significantly upregulated in Natural killer (NK) cells-derived exosomes from lean mice, which directly targets SKI family transcriptional corepressor 1 (SKOR1), subsequently downregulated the expression levels of pro-inflammatory cytokine factors (including IL-1β, IL-6, and TNF-α) levels and attenuated IR. Therefore, it might be that metabolism-regulating miRNAs play a vital role in the dynamics of metabolic homeostasis.^256^

#### LncRNAs and IR

Long non-coding RNAs (lncRNAs)^257^ are non-coding transcripts more than 200 nucleotides, and the subcellular localization of lncRNAs determines their function. LncRNAs located in the nucleus could affect chromosomal biology or interact with transcription factors to regulate gene transcription; lncRNAs located in cytosol could modulate mRNA stability and translational efficiency by acting as sponges for miRNAs or direct pairing with mRNA. Recent advances have shown that lncRNAs play crucial roles in the pathologys of IR and diabetes.^213,258,259^

Glucose and lipid metabolism disorders are the primary causes for the pathophysiological development of IR. The lncRNA SRA promotes insulin-stimulated glucose uptake by co-activating PPARγ, leading to increased phosphorylation of the downstream targets Akt and FOXO1 in adipocytes.^260^ Furthermore, glucagon-stimulated upregulation of H19 via the AMP/PKA pathway induces nuclear translocation of HNF4A and activates the transcription of *G6PC* and *PCK* that are involved in gluconeogenesis, resulting in hepatic glucose production.^261^ In addition, the H19 sponge cell miR-130a induces PPARγ nuclear translocation, thereby activating the transcription of adipogenic genes like those encoding acetyl coenzyme a carboxylase 1 (ACC1), fatty acid synthase (FAS), and cytochrome c oxidase (SCO1), thereby promoting intracellular lipid accumulation.^262^ H19 is downregulated in skeletal muscles of db/db mice and interacts with heterogeneous nuclear ribonucleoprotein (hnRNPA1) that then increases fatty acid oxidation (FFA) protein translation. These processes are closely related to the genes *PGC1a* and *CPT1b* that reverse FFA-induced lipid accumulation and improve IR.^263^ This suggests the complex effect of lncRNAs on the IR progression.

In addition the insulin target tissues,^264^ transcriptome profiling and different studies have identified several β-cell specific lncRNAs that contribute to obesity-mediated β-cell dysfunction and apoptosis. LncRNA MALAT1 downregulation may lead to pancreatic β-cell dysfunction and T2DM development by direct interaction and regulation of polypyrimidine bundle binding protein 1 (PTBP1). The lncRNA-p3134 positively regulates GSIS by promoting PI3K/Akt/mTOR signaling and the key regulators (Pdx-1, MafA, GLUT2, and Tcf7l2) in pancreatic β cells. Further, lncRNA-p3134 overexpression can decrease the β cell apoptosis ratio and partially reverse the glucotoxicity effects on GSIS function.^265,266^ Similarly, the newly identified lncRNA β-cell function and apoptosis regulator (βFaar) ameliorates obesity-associated β-cell dysfunction and apoptosis by upregulating the islet-specific genes (*Ins2*, *NeuroD1*, and *Creb1*) by sponging miR-138-5p.^267^ In addition, a novel micropeptide TUNAR encoded by lncRNA transcripts play a critical role in pancreatic β cell functions and insulin homeostasis.^268^ Collectively, these studies provide new insights into the use of lncRNAs as possible biomarkers or therapeutic targets for obesity-associated IR and metabolic diseases.

#### Circular RNAs (circRNAs) and IR

Contrary to conventional linear RNA, circRNAs are noncoding RNAs that generated from precursor mRNAs by back-splicing circularization, which is derived from exonic circRNAs, intronic circRNAs, exonic-intronic circRNAs and ntergenic circRNAs.^269^ CircRNAs can affect gene transcription, splicing and translation by acting as a miRNA sponges, binding to RNA binding proteins or transcription factors (TFs). Recent studies have suggested that newly identified circRNAs are novel factors in the initiation and development of IR.^270^ For example, ci-Ins2 is a conserved intronic circRNA derived from insulin pre-mRNA that exhibits lower levels in the islets of rodents and humans with type 2 diabetes.^271^ ci-Ins2 silencing in pancreatic islets leads to decreased expression of several genes important for insulin secretion (*Rapgef4*, *Pld1*, *Pclo*, and *Cacna1c*) by interacting with the TAR DNA-binding protein 43 (TDP43), thereby contributing to β-cell dysfunction during diabetes. CircHIPK3 is one of the most abundant circRNAs in β-cells and regulates hyperglycemia and IR by sequestering miR-124-3p and miR-338-3p, thereby increasing mRNA expression of key β-cell genes (e.g., *Slc2a2*, *Akt1*, and *Mtpn*), insulin secretion, and β-cell proliferation.^272^ A similar effect of circHIPK3 on hyperglycemia and IR has been observed by sponging miR-192-5p and increasing FOXO1 expression^273^. In addition, Hsa_circ_0054633 suppression promotes β-cell proliferation and facilitates insulin secretion through inhibiting caspase-8 expression by sponging miR-409-3p.^274^ These recent results point to circRNAs as novel regulators of β-cell dysfunction under diabetic conditions.

Similar to the miRNAs and lncRNAs, several circRNAs also contribute to the the regulation of glucose and lipid homeostasis.^275^ Li et al.^276^ first demonstrated that circRNA-1897 is highly downregulated in the subcutaneous tissues of two pig breeds, and that it directly targets miR-27a and miR-27b-3p that are negative regulators of adipocyte differentiation by suppressing PPARγ expression. Deep sequencing analysis of adipose circRNA revealed that circArhgap5-2 is highly upregulated during differentiation of human white adipocytes.^277^ circArhgap5-2 silencing results in inhibited lipid accumulation and adipose marker (PPARγ, AdipoQ Cebpα, and FABP4) downregulation. Thus, circRNAs likely serve as important regulators of adipocyte differentiation and lipid metabolism. Another circRNA deep sequencing analysis of sera from patients with metabolic syndrome (MetS) identified the presence of a novel circRNA, circRNF111, involved in MetS progression.^278^ CircRNF111 inhibition enhances IR and lipid deposition in MetS by regulating the miR-143-3p/ IGF2R pathway.

AMPK is a critical factor in energy homeostasis including glycolysis, lipolysis, and fatty acid oxidation (FAO). CircACC1 is a circRNA derived from the human acetyl-CoA carboxylase 1 (*ACC1*) gene and directly binds to the β and γ subunits of AMPK, facilitating its activity,^279^ and promoting glycolysis and fatty acid β-oxidation during metabolic stress. circMAP3K4 is another potentially important circRNA involved in glucose metabolism that is highly expressed in the placentas of patients with gestational diabetes mellitus (GDM) and the IR model.^280^ circMAP3K4 can suppress the insulin-PI3K/Akt signaling pathway via the miR-6795-5p/PTPN1 axis, thereby contributing to GDM-associated IR. Nevertheless, the exact roles and regulatory mechanisms of circRNAs in IR require additional clarity.

### Involvement of the gut microbiota in IR

#### Influencing factors of Gut microbiome composition

The microbes living in the human gut are key contributors to host metabolism and immune function through mediating the interaction between the host and environment, or releasing metabolites and cytokines.^281^ In 2012, the Human Microbiome Project Consortium began to show that the gut microbial phyla in humans mainly consist of the gram-positive Firmicutes, gram-negative Bacteroidetes and Proteobacteria.^282^ Although the composition of human gut microbiota remains relatively stable from around age 3, gut microbiota undergoes the increase in diversity and altered proportions of composition.^283^

Different factors influencing these alterations of gut microbiome composition have been explored including diet, exercise, circadian disruption, antibiotics treatments, and genetics.^284^ (1) Regarding diet: David et al.^285^ conducted a study of human wherein volunteers were placed on either a plant-based diet (i.e., with grains, legumes, fruits, and vegetables) or an animal product-based diet (i.e., with meats, eggs, and cheeses) for five consecutive days. The gut microbial communities of the groups significantly diverged over time, with participants on animal diets experiencing proliferation of bile-tolerant microorganisms (e.g., *Alistipes*, *Bilophila*, and *Bacteroides*) and decreased abundances of fiber-fermenting bacteria. Furthermore, differences in gut microbiota exists between humans with western diets rich in lipids and animal proteins in comparison to African diets rich in millet/sorghum and local vegetables, with little contribution of lipids and animal proteins to diets.^286^ (2) With regards to exercise, recent studies have highlighted the capacity of exercise to increase the abundances of beneficial gut microbial species, increasing gut microflora diversity, improving the proliferation of commensal bacteria, and reducing inflammation in addition to intestinal permeability.^287,288^ (3) Circadian disruption: both human and non-human models examination indicate that insufficient sleep (less than 7 h sleep per night) and circadian misalignment (such as workforce with shift workers or social jetlag) may lead to modifications in gut microbial diversity, structure and function.^289,290^ (4) Antibiotics: In deed, short-time antibiotic exposures can directly perturb the gut microbiota, reduce bacterial diversity and metabolic activity, disrupt intestinal integrity,^291^ which is a major cause for concern in human health. (5) Host genetics also shape the composition of the gut microbiome. For example, microbiome genome-wide association studies (mGWAS) have identified that variants of different genes (for example, *VDR*, *LCT*, *NOD2*, *FUT2*, and *APOA5*) that are associated with distinct gut microbiome compositions.^292^ Furthermore, 16 S ribosomal RNA (16 S rRNA) sequencing with microbiome analysis revealed that some species (especially from the phyla Firmicutes and Verrucomicrobia) in the gut microbiome are heritable.^293^ Thus, how to modulate the gut microbiota based on internal and external factors is important to maintain the public health.

#### Gut microbiome dysbiosis involved in IR

Growing evidence in the last two decades has suggested that gut microbial dysbiosis contributes to increased risks of metabolic defects like obesity, IR, and diabetes.^294^ For example, several studies have shown that obese adults harbor reduced gut microbial diversity and altered microbiota compositions compared with adults exhibiting normal weight.^295^ Another study of sixty-eight obese young patients revealed reduced fecal bacterial richness in patients with IR and high diastolic blood pressure (BP).^296^ Moreover, distinct microbial population markers were associated with impaired glucose tolerance, high BP, and low high-density lipoprotein cholesterol.^297^ A whole-genome sequencing investigation of the intestinal microbiota from 49 obese adults revealed that low bacterial gene counts were associated with unhealthy phenotypes like higher IR, dyslipidemia, and inflammation compared to adults with higher bacterial gene counts.^298^ While the exact roles of gut microbiomes in IR remain incompletely understood, many studies have nevertheless begun to elucidate the mechanisms by which gut microbiome dysbiosis produces different signaling activation.^299^ For example, gut microbiota can influence host glucose metabolism and hormone production via the production of several metabolites like short-chain fatty acids (SCFAs, mainly including acetate, propionate, and butyrate) and bile acids.^300^ Hyperglycemia then increases gut permeability and subsequent translocation of bacterial lipopolysaccharide (LPS) into systemic circulation. LPS circulation then contributes to the chronic inflammation of liver and adipose tissue that is associated with the development of IR, in addition to other conditions associated with metabolic syndromes.^301^ The potential mechanisms related to gut microbiome activities and IR are very complex, and numerous studies with contradictory results render it difficult to identify clear mechanistic pathways. Nevertheless, some strategies have been developed to modulate microbiota, such as fecal microbiota transplants, probiotics or prebiotics supplementation, in combination with medications and/or healthy lifestyle, in hope to ameliorate microbiota composition and IR.^302,303^

## IR related diseases in human

**Fig. 4:**
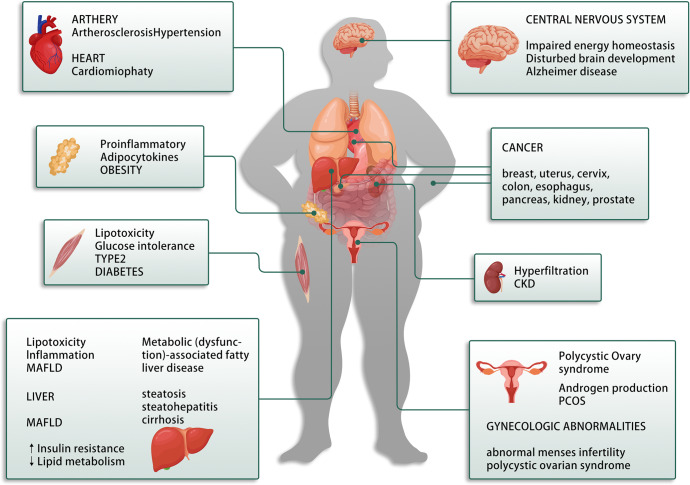
Insulin resistance related diseases in human

As we all know, IR is a state in which higher than normal concentrations of insulin are needed for a normal response, leading directly to hyperinsulinaemia and impaired glucose tolerance.^304^ As mentioned above, the primary characteristics of IR are inhibited lipolysis in adipose tissue, impaired glucose uptake by muscle and inhibited gluconeogenesis in liver.^305^ Nevertheless, IR can be linked to a cluster of abnormal syndrome (Fig. 4), which include obesity, diabetes, Nonalcoholic fatty liver disease, cardiovascular disease, polycystic ovary syndrome, and other abnormalities.^306–308^ Since obesity and diabetes have been discussed in the previous content, this part we mainly summary other related metabolic syndrome in human.

### Metabolic (dysfunction)-associated fatty liver disease (MAFLD) and IR

Non-alcoholic fatty liver disease (NAFLD) is one of the most common liver diseases worldwide.^309^ It includes a series of diseases, such as simple fatty liver (hepatic steatosis), non-alcoholic steatohepatitis (NASH), liver cirrhosis, and hepatocellular carcinoma.^310^ Recently, a consensus of international experts proposed that the disease acronym have to be changed from NAFLD to metabolic (dysfunction) associated fatty liver disease (MAFLD). Adipose tissue is a physiologic reservoir of fatty acids,^311^ when the storage capacity is exceeded, the accumulation of heterotopic lipids leads to lipotoxicity, thereby promoting low-grade inflammation and IR in the liver.^312^ Lipotoxicity impairs insulin signaling, induces oxidative damage, promotes inflammation and fibrosis,^313^ which is thought to be related to the progression of MAFLD patients from simple steatosis to NASH, hepatic fibrosis, and hepatocellular carcinoma. Lipotoxic injury appears to occur in response to excessive levels of serum free fatty acids (FFAs) in hepatocytes.^314^ Circulating FFAs are the primary causes of liver fat accumulation in MAFLD that mainly occur due to lipolysis of adipose tissues, and partially from excess lipoprotein.^315^ Patients with MAFLD have increased levels of FFAs owing to a failure of insulin-mediated suppression of lipolysis, and excess FFAs excretion into the bloodstream.^314^ In addition to the influence of abnormalities on lipid metabolism, IR also indirectly contributes to MAFLD through inflammation.^316,317^ The transcription factor NF-κB exhibits higher levels in liver and adipose tissues during IR.^318^ Further, activation of NF-κB translocation to the nucleus leads to upregulated of the expression of target genes that encoded inflammatory mediators, like TNF-α and IL-6, which are released by hypertrophic adipocytes and elevated during MAFLD.^319^ In addition, adiponectin is an anti-inflammatory adipokine that mediates fatty acid β-oxidation (FAO), glucose use, and suppression of fatty acid synthesis.^320,321^ Hepatic adiponectin expression is lower in NASH patients, while the levels of adiponectin and its receptors increase after weight loss.^322^ Moreover, adiponectin overexpression increases subcutaneous fat levels and protects against diet-induced IR.^323^ Isotopic tracer studies have shown that MAFLD patients exhibit increased de novo lipogenesis (DNL), that is respectively mediated by two transcription factors SREBP-1c and ChREBP.^324,325^ SREBP-1c is a major regulator of fatty acid synthesis and hepatic SREBP-1c overexpression increases DAG content and PKCε translocation that in turn impairs INSR tyrosine kinase activity, thereby inducing hepatic IR.^326,327^ While ChREBP deficiency improves IR and hepatic steatosis by inhibiting the entire lipogenic and esterification process.^328,329^ Thus, inhibition of DNL and related pathway may effectively alleviate MAFLD and IR.

### Polycystic ovary syndrome (PCOS) and IR

Polycystic ovary syndrome (PCOS) is an endocrine and metabolic disorder characterized by imbalances of multiple hormones that reflect the clinical manifestations of hyperandrogenism and affects 5%–10% of women of childbearing age.^330,331^ It is believed that IR and obesity play prevalent roles in causing PCOS, and PCOS women show an higher increased comorbidities of IR, including obesity, dyslipidemia, hypertension, and T2DM than healthy women.^332–334^ Specifically, IR leads to compensatory hyperinsulinemia, which stimulates GnRH gene transcription through MAPK pathway in PCOS and increases LH pulse secretion, thereby significantly increasing ovarian androgen synthesis.^12,332^ In addition to directly interfering with insulin signaling, androgens may also trigger lipolysis and increase circulating FFA, thereby leading to IR.^335^ Moreover, androgens decrease the type I muscle fibers (TIMF) with highly oxidative and insulin-sensitive properties, while increase type II muscle fibers (TIIMF) that are glycolytic and less insulin-sensitive, further decreasing glycogen synthase expression and favoring the development of IR in PCOS.^336–338^ This evidence supports that IR and hyperandrogenemia continuously stimulates each other in a vicious cycle under the condition of PCOS. At present, the molecular mechanism of insulin in PCOS has been well described. First of all, the defects downstream of insulin receptor phosphorylation, such as activation of phosphorylated IRS-1 through PKC or GLUT-4 translocation through PI3K/Akt signaling pathway, are the causes of IR in some PCOS women.^41,339^ Second, certain proinflammatory mediators including TNF, C-reactive protein (CRP), monocyte chemoattractant protein-1 (MCP-1) and IL-18 levels are elevated in PCOS women independently of obesity.^340–342^ Furthermore, hyperglycemia may contribute to inflammation in PCOS, possibly by inducing oxidative stress via increased ROS production. Such modifications then activate NF-κB that is involved in the expression of proinflammatory mediators such as TNF and IL-6,^343,344^ and that induces key steroidogenic molecules, like CYP11A1, CYP17A1 and StAR, leading to further aggravation of hyperandrogenemia.^345–347^ Altogether, obesity and IR play pivotal role in women with PCOS and subsequent metabolic complications, and targeting these areas may become an important therapeutic approach for effectively reducing incidence of this pathology.

### Cardiovascular disease and IR

Cardiovascular diseases (CVDs) are the leading causes of death globally. The World Health Organization estimates that 17.9 million people live with CVDs each year, and CVD-related deaths accounted for 32% of all global deaths in 2019. Moreover, over 23 million people are estimated to die from CVDs each year by 2030.^348–350^ CVDs represent a general compromising abnormal conditions including any disorders of heart and blood vessels. However, the most common types of CVDs include high blood pressure, coronary artery disease (CAD), stroke, cerebrovascular disease and rheumatic heart disease (RHD).^351,352^ Currently, the mechanisms of IR contribute to cardiovascular diseases mainly include chronic hyperglycemia, dyslipidemia, endothelial dysfunction and inflammation.^9,353–355^ Specifically, the increased gluconeogenesis and decreased glycogen synthesis in hepatic IR results in fasting hyperglycemia that increases total TG levels and blood pressure (BP), reduces HDL-C levels, and increases the risk of thrombosis formation.^43,356^ Moreover, long-term follow up data from patients with type 1 and type 2 diabetes have confirmed that hyperglycemia is a risk factor for CVD.^357^ IR in adipose tissue leads to high FFAs levels,^358^ visceral fat accumulation that is associated with elevated levels of plasminogen activator inhibitor 1 (PAI-1) and BP,^359^ and ectopic lipid and toxic lipid metabolite accumulation (lipotoxicity) in blood vessels that alters cellular signaling and cardiac structure, thereby contributing to the increased prevalence of cardiovascular diseases.^360,361^ Furthermore, IR induces dyslipidemia characterized by elevated serum total cholesterol (TC), low-density lipoprotein cholesterol (LDL-C), or triglycerides (TG) along with reduced HDL-C concentrations, together which enhance the incidence of CVD by 32% in men and 76% in women.^362,363^ IR contributes to endothelial dysfunction by decreasing nitric oxide (NO) production via PI3K/Akt pathway from endothelial cells,^364^ and increasing reactive oxygen species (ROS) production, prothrombotic factors and proinflammatory markers mediated by MAPK/ERK activity,^365^ that both increases the cardiovascular risk, increased ROS levels in turn leads to the inhibition of insulin-PI3K signaling pathway through IRS-1 phosphorylation, which may aggravate IR.^366^ Overall, IR is a complex syndrome, which can significantly increase the risk of cardiovascular diseases. Identifying new therapies to reduce IR may contribute to the reduced prevalence of CVDs.

### Alzheimer’s disease and IR

Alzheimer’s disease (AD) is a progressive neurodegenerative disease and is considered the sixth leading cause of death in the United States, with most patients aged 65 or older.^367^ Recent epidemiological studies have suggested that IR increases the risk for AD and related dementias.^368^ AD is actually a brain-specific form of diabetes that exhibit increased Aβ accumulation, tau hyperphosphorylation and impaired glucose transportation, energy metabolism, hippocampus and inflammatory pathways.^369^ Additional research has identified that insulin receptor is expressed on almost all cell types in the brain, with expression highest in the olfactory bulb, followed by the cerebral cortex, hippocampus, hypothalamus, and cerebellum.^370–372^ Thus, insulin signaling likely also carries important and diverse roles in brain functioning and AD pathogenesis. Insulin primarily enters the brain via selective, saturable transport across the blood-brain barrier (BBB)^373–375^ Peripherally produced insulin can also be actively transported into the brain via an endocytic-exocytic mechanism.^376^ Similar to systemic IR, brain IR can be defined as failed response to insulin by brain cells,^377,378^ primarily due to downregulated insulin receptors, an inability of insulin receptors to bind insulin, or dysfunction of the insulin signaling cascade.^379^

Current researches have demonstrated that the mechanisms of systemic IR and brain-specific IR have close links with AD pathogenesis. For example, major abnormalities in AD brains include decreased mRNA and protein expression levels of insulin, insulin receptors, insulin receptor substrate 1 (IRS1) and IGF1/2, in addition to reduced protein indicators of downstream insulin signaling activity (including phosphorylated AKT (pAKT) and phosphorylated GSK3β), tau mRNA, and increased amyloid precursor protein levels.^380,381^ Furthermore, recent evidence indicated that inflammation and lipid metabolism might contribute to the development of AD.^382^ Potential targets include PPARγ, Apoliprotein E (ApoE), Apolipoprotein E receptor (LRP1), and leptin.^383–385^ Chronic inflammation exacerbates IR signaling that contributes to AD by provoking proinflammatory mediators including TNF-α, IL-6, and IL-1β.^386–389^ Among these mediators, IL-6 can stimulate amyloid precursor protein (APP) formation, and is often co-localized with Aβ plaques in AD patients.^390–392^ Thus, rosiglitazone has anti-inflammatory effect by decreasing levels of NFκB and inhibiting the Aβ42 production in mice, is considering as therapeutic agent for AD. Pioglitazone acts similarly as Rosiglitazone by reducing tau and Aβ deposits in the hippocampus, and improving neuronal plasticity and learning in AD.^393–395^ These studies collectively suggest that IR contributes to AD pathogenesis through multiple pathways. Moreover, overlapping pathological features exist for diabetes, IR, and AD. Thus, the development of additional therapeutic drugs including antidiabetics or IR interventions with beneficial effects against cognitive impairment and Alzheimer’s disease carry promising future application potentials.

### Chronic kidney disease and IR

Chronic kidney disease (CKD) involves a gradual loss of kidney function and inability to filter blood^396,397^ and is a major risk factor for end-stage kidney failure (ESKF) and CVDs.^398,399^ And the inflammatory and glycometabolic abnormalities that closely related to IR are common features in CKD and CVD, which explain the strong relationship between them.^400^ In the last decades, researchers have showed that IR is an early metabolic alteration in CKD, because IR plays a primary role in metabolic syndromes characterized by abdominal obesity, high fasting glucose levels, hypertriglyceridemia, depressed serum HDL-C, and high blood pressure, that are commonly observed in CKD patients.^401–403^ Furthermore, CKD patients demonstrate systemic inflammation and elevated levels of pro-inflammatory cytokines like C-reactive protein (CRP), TNF-α, IL-6 and IL-1β.^404,405^ In particular, reduced renal excretion leads to abnormal plasma adipokines levels including leptin and adiponectin in CKD patients.^406^ Leptin may also be considered as a uremia toxin through proinflammatory effects,^407,408^ while adiponectin mediates insulin-sensitizing and anti-inflammatory responses.^409–411^ Indeed, an accumulation of leptin is larger than adiponectin in CKD, and this abnormal ratio may further promote IR and metabolic disorders.^412^ Despite these above factors, evidences persist that endothelial dysfunction, oxidative stress, and vitamin D deficiency are important in the glucose intolerance pathogenesis and IR in patients with CKD.^413–415^ Thus, newly developed methods for improving IR could lead to potential strategies for preventing excess mortality of CKD patients.

### Cancer and IR

Numerous recent epidemiological studies have suggested that IR increases the risks for different cancers including colon, liver, pancreas, breast, endometrium, thyroid and gastric cancer.^416–418^ Diverse cellular and molecular mechanisms are involved in the relationship between IR and cancer. Further, a growing body of evidence suggests that increased insulin, in addition to IGF1 and IGF2 levels critically influence tumor initiation and progression in IR patients.^419^ Specifically, the three ligands (insulin, IGF1, and IGF2) binds the receptors (IGF-IR and INSR) and activate the insulin receptor substrates. This in turn, first activates the PI3K/Akt/mTOR, PI3K/Akt/FoxO, or Ras/MAPK/(ERK-1/2) pathways that have important roles in cancer cell growth and carcinogenesis.^420–422^ Second, these processes inactivate GSK3β through the PI3K/Akt signaling pathway, resulting in oncogenic β-catenin signaling activation, that has been associated with cancer stemness and chemoresistance.^423,424^ In addition, insulin and IGF1 inhibit sex-hormone binding globulin (SHBG) synthesis, although both hormones stimulate ovarian synthesis of sex steroids that can promote cellular proliferation and inhibit apoptosis in breast epithelium and endometrium.^416,425^ Furthermore, the increased risk of cancer in IR patients may be due to excessive ROS production that then impairs the contribution of DNA to mutation and carcinogenesis.^426–428^ With the elucidation of more new molecular mechanisms of IR and cancer, the relationship of IR with different tumors will be more complicated, and novel diagnostic and therapeutic strategies may provide a new approach for preventing cancer other related diseases.

## The diagnosis and therapeutic strategy of IR

### Diagnosis methods of IR

**Fig. 5:**
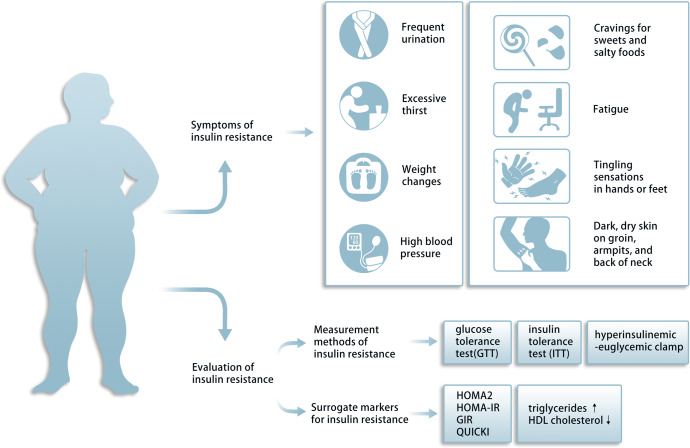
Ex vivo diagnosis methods for insulin resistance

As we all know, IR is related to several metabolic abnormalities including obesity, glucose tolerance, dyslipidemia, type 2 diabetes and other metabolic syndrome.^429,430^ Several methods are used to measure blood insulin levels that primarily include glucose tolerance tests (GTTs), insulin tolerance tests (ITTs), hyperinsulinemic-euglycemic clamp (HEC), continuous infusion of glucose with model assessment (CIGMA), the minimal model technique (MMT), insulin suppression test (IST), and insulin release tests (IRTs) (Fig. 5), and their differences are the sensitivity, limitation, and complexity of technical procedures.^35,431^ Glucose tolerance test (GTT) is given to determine how quickly exogenous glucose delivered via oral, intraperitoneal, or intravenous administration is cleared from the blood.^432^ The GTT method is used to diagnose diabetes mellitus including T1DM, T2DM and GDM.^433^ The Insulin Tolerance Test (ITT) is designed to examine the systemic sensitivity of insulin receptors by measuring blood glucose levels changes before and after intravenous insulin administration.^434^ This method is used to assess the insulin-sensitizing efficacy of test compounds and pharmacological agents that can modify insulin sensitivity.^435^ However, ITT often induces adequate hypoglycemia, severe hypokalaemia, it may as the systemic counter regulatory responses following the intravenous insulin.^436,437^ Despite these limitations, GTT and ITT are the most widely tests for assessing insulin sensitivity, largely because they are inexpensive and easy to perform.^438^ The HEC has been considered as the gold-standard method to assess insulin sensitivity in vivo. Actually, IR precedes the occurrence of T2DM, so how to increase the accurate assessment of insulin sensitivity is very important to predict the risk and evaluate the management of impaired insulin sensitivity and metabolic syndrome in research and clinical practice.

As the same time, some other evaluation indices have been developed and tested the insulin sensitivity/resistance. HOMA2 (updated HOMA model which took account of variations in hepatic and peripheral glucose resistance), homeostatic Model Assessment for IR (HOMA-IR), the oral glucose insulin sensitivity index (OGSI), fasting Insulin (FINS), and fasting plasma glucose (FPG) based on fasting glucose and insulin levels^439–443^ are widely utilized IR measurements in clinical research. Other indices based on fasting insulin include the glucose to insulin ratio (GIR), the quantitative insulin sensitivity check index (QUICKI),^444–446^ triglycerides (McAuley Index) alone or in accordance with HDL cholesterol (HDL-C),^447^ whole-body insulin sensitivity index (WBISI), Matsuda Index to evaluate whole body physiological insulin sensitivity by the above methods. Indeed, the early symptoms of IR in different individuals are not obvious, and the related symptoms are very complex, combining with screening indicators may provide more precise diagnosis for IR in the general population.

### Therapeutic strategy of IR

#### Clinical approved treatment to IR

**Fig. 6:**
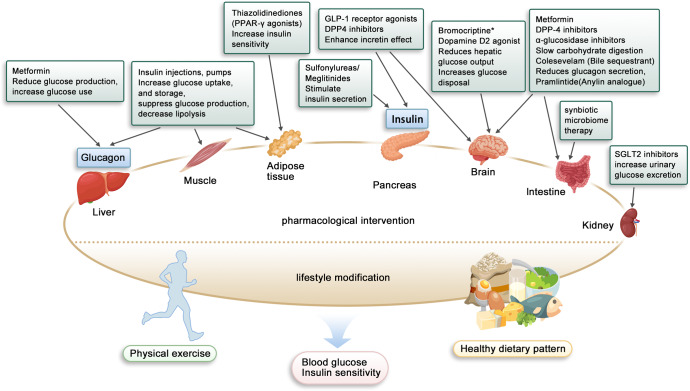
Therapeutic strategy of insulin resistance

No medications exist currently that are specifically approved to treat IR, but IR management^91,448,449^ is possible through lifestyle changes like dietary, increased exercise, and disease prevention in addition to alternative medications (Fig. 6). Among these treatments, lifestyle changes should be the main focus for IR treatment, with nutritional intervention to decrease calories, avoidance of carbohydrates, and focusing on aliments with low glycemic index (including vegetables, fruits, whole-grain products, nuts, lean meats or beans) to provide higher fiber, vitamins, healthy fats and protein are particularly helpful for people trying to improve insulin sensitivity.^450–452^ A healthy diet and regular physical exercise including approximately 30 minutes of exercise at least five days a week leads to activation of muscle cells that increase AMPK activity, thereby inactivating TCB1D1 and promoting GLUT4 translocation to cellular membrane, in addition to increasing glucose uptake that increases insulin reactivity.^453,454^ Moreover, losing just 5%–7% of body weight can prevent or delay 60% of diabetes and ameliorate insulin sensitivity in obese and overweight individuals.^455^ Some specific pharmacological medications have been used as a preventives in type 2 diabetes by improving insulin sensitivity, primarily including Biguanides, Thiazolidinediones and GLP-1 receptor agonists, etc. (Table 1). Metformin is a first-line medication and the most widely-prescribed insulin-sensitizing agent in T2DM and PCOS patients.^456,457^ Metformin mediates improved insulin sensitivity by increasing insulin receptor tyrosine kinase activity, enhancing glycogen synthesis, and increasing the recruitment and activity of the glucose transporter GLUT4.^458–460^ Metformin also promotes re-esterification of free fatty acids and inhibits lipolysis, which may then indirectly increase insulin sensitivity by reducing lipotoxicity in adipose tissues.^461^ In addition to metformin, other targeted drugs exist for T2DM treatment and improving insulin sensitivity. For example, (1) Glucagon-like peptide 1 (GLP1) is an intestinal hormone that can enhance insulin secretion in a glucose-dependent manner by activating the GLP-1 receptor (GLP-1R) that is highly expressed on islet β cells.^462,463^ GLP-1 receptor agonists including Liraglutide, Semaglutide, Dulaglutide and Exenatide have now been approved for treating T2DM.^464–468^ These GLP-1 receptor agonists can suppress the inflammatory response of macrophages, thereby inhibiting IR.^469^ (2) Dipeptidyl peptidase-4 (DPP-4) also referred as the T-cell antigen CD26 can degrade GLP-1.^470^ DPP is a local mediator of inflammation and IR in adipose and hepatic tissue that interacts with the integral membrane protein, caveolin-1, then impairing the activation of down-stream AKT signaling.^471–473^ DPP-4 inhibitors (such as gemigliptin, saxagliptin, sitagliptin, teneligliptin, trelagliptin, vildagliptin, et al.) are now world-wide therapy of T2DM since 2006 and could improve insulin sensitivity.^474–476^ (3) A third targeted drug is sodium-glucose cotransporter (SGLT2) that is the major cotransporter involved in glucose reabsorption in kidneys.^477^ SGLT2 inhibitors include canagliflozin, dapagliflozin, and empagliflozin that have been approved by the FDA for combined use with diet and exercise to lower blood sugar and excessive insulin secretion in adults with type 2 diabetes.^478–480^ In addition, SGLT2 inhibitors exhibit beneficial effects for reducing IR and protecting pancreatic β cell functioning.^481,482^ (4) A fourth targeted drug approach is the activation of PPAR-γ in mature adipocytes that then induces altered expression of several genes (increased GLUT4 and adiponectin expression, along with decreased TNF-α, IL-6 and leptin expression) involved in the insulin signaling cascade, thereby improving insulin sensitivity.^483–485^ PPAR-γ agonists (thiazolidinediones and TZDs) used for diabetes mellitus, which enhance adipocyte lipid storage, decrease ectopic lipid accumulation and improve insulin sensitivity in liver and skeletal muscles.^486,487^ Unfortunately, these agents have produced limited success due to reduced efficacy, low tolerability, and significant side effects including hypoglycemia, weight gain, bone fractures, and vomiting.^488,489^ Thus, it is urgent to find new approaches to treat T2DM and modify insulin sensitivity.

**Table 1:** Clinical medication for improving insulin resistance

Type	Listed drugs	Mechanism
Biguanides	Metformin	The exact mechanism of metformin is still unclear and may be related to increased insulin receptor tyrosine kinase activity, enhanced glycogen synthesis, and recruitment of the GLUT4 glucose transporter.
Thiazolidinediones	Pioglitazone	It mainly activates peroxisome proliferator-activated receptor γ (PPAR-γ) to enhance the sensitivity of adipose muscle and liver to insulin.
	Rosiglitazone	
GLP-1 receptor agonists	Liraglutide	GLP1 receptor agonists (GLP-1RAs) can affect IR by increasing the expression of glucose transporters in insulin-dependent tissues, reducing inflammation and oxidative stress, and regulating lipid metabolism.
	Exenatide	
	Semaglutide	
	Dulaglutide	
DPP-4 inhibitors	Saxagliptin	It can decrease the degradation of GLP-1 by inhibiting the activity of DPP4, thereby exerting a role in the treatment of type 2 diabetes.
	Vildagliptin	
	Alogliptin	
	Linagliptin	
	Gemigliptin	
	Teneligliptin	
	Trelagliptin	
Sulfonylureas	Glimepiride	It promotes insulin receptor activation, thereby increasing the amount of glucose transporters, which in turn increases insulin sensitivity and improves insulin resistance.
PPAR full agonists	Chiglitazar Sodium	Chiglitazar Sodium is a peroxisome proliferator-activated receptor (PPAR) full agonist that simultaneously activates three subtypes of PPAR receptors (α, γ, and δ). It can induce the expression of downstream target genes related to insulin sensitivity, fatty acid oxidation, energy conversion and lipid transport, and inhibit the phosphorylation of PPARγ receptors associated with insulin resistance.

#### Clinical trials for insulin sensitivity management

In clinical research, scientists and physicians have explored different strategies to prevent and treat diabetes mellitus and IR. We have searched the complete clinical trials (https://clinicaltrials.gov) to reduce IR and summarized them mainly include: (1) Diet intervention, such as Low-fat vegetarian Food, high-protein food, calorie restriction, vitamin D supplementation to reduce the IR in human obesity.^490,491^ (2) Pharmacological Intervention, such as BFKB8488A, the anti-fgfr1/KLB agonist antibody mimics the effect of FGF21, and causes short-term weight loss and increases insulin sensitivity.^492,493^ Several studies have also demonstrated that chromium picolinate administration lowers glucose and insulin levels in patients with type 2 diabetes;^494,495^ Salsalate inhibits IKK/NF-kB and may improve insulin sensitivity.^496^ Besides the above drugs, structural and functional dysbiosis of intestinal microbiome are induced in obese rodents,^497,498^ and they may cause IR and systemic inflammation.^294,499,500^ Thus, synbiotic therapy on intestinal microbiota has been developed as a new treatment strategy in clinical study (NCT04642482). We present some clinical trials of IR intervention in Table 2. Over the past years, our knowledge of the pathogenesis of IR and T2DM has improved, the development of new treatments of IR and metabolic syndrome have gained certain success, while the complexity of IR and the presence of multiple feedback loops make a challenge to the specific intervention.

**Table 2:** Selected clinical trials for insulin resistance in recent ten years (2013–2022)

Conditions	Starting time	Phase	Interventions	Treatment schedule	Outcome measures	NCT Number
HIV Related Insulin Resistance|Protease Inhibitor Related Insulin Resistance|Endoplasmic Reticulum Stress	2013	Not Applicable	Drug: Tauroursodeoxycholic acid	The intervention group will receive 1.75 g of tauroursodeoxycholic acid daily for 30 days.	Glucose Uptake|Body Composition|Liver Fat| Liver Function Tests	NCT01877551
Metabolic Syndrome X	2013	Phase 3	Drug: Ezetimibe	Ezetimibe 10 mg/d for 12 weeks	Change in Intestinal mRNA Expression Levels of LDL Receptor |Change in Intestinal mRNA Expression Levels of SREBP-2, NPC1L1, ABCG5/8, PCSK9 and HMG CoA	NCT01849068
Type 2 Diabetes Mellitus	2015	Phase 4	Drug: Dapagliflozin	Dapagliflozin 10 mg Tablets, Oral, Once Daily, 8 weeks	Adjusted Change From Baseline in Skeletal Muscle Insulin-stimulated Gluocose Uptake|Adjusted Change in Adipose Tissue Insulin-stimulated Glucose Uptake|Adjusted Change in Liver Insulin-stimulated Glucose Uptake From Baseline to Week 8	NCT02426541
Diabetes|Metabolic Syndrome|Insulin Resistance	2013	Phase 2	Drug: Pegvisomant	Pegvisomant 20 mg subcutaneously Qday will be administered by the study subject for 28 days during this study.	Insulin Sensitivity|Lipolysis	NCT02023918
Insulin Resistance |Obesity|Sedentary Lifestyle	2013	Not Applicable	Behavioral: Exercise training	Study participants in the intervention arm of the study will be asked to exercise on 3 days per week for 48 weeks.	Change From Baseline in Volume of Exercise (Total Time)|Insulin Resistance|Exercise Fitness|Body Composition	NCT01848353
Diabetes Mellitus, Type 2|Skeletal Muscle Insulin Sensitivity	2017	Phase 4	Drug: Dapagliflozin	Patients will receive dapagliflozin 10 mg in tablet for a maximum of 40 days	Corrected Glucose Disposal Rate (cGDR) Measured as Change in Rate of Disposal (Delta RD) Basal vs High Insulin After 5 Weeks of Treatment	NCT03338855
Glucose Intolerance	2015	Not Applicable	Dietary Supplement: Artemisia dracunculus	Artemisia Dracunculus extract, 2 capsules of 500 mg, two times per day before breakfast and dinner during 90 days	Postprandial Glucose Levels |Fasting Glucose Levels |Glycosylated Hemoglobin |First Phase of Insulin Secretion|Total Insulin Secretion|Insulin Sensitivity|	NCT02330341
Diabetes Mellitus, Type 2	2018	Not Applicable	Behavioral: SLEEP-Extend intervention	The subjects extended their sleep time by at least 1 hour but not more than 2 hours for 4 weeks.	Homeostatic Model Assessment for IR (HOMA-IR) Scores	NCT03616171
Metabolic Syndrome	2014	Phase 4	Drug: Sildenafil citrate	Subjects took Sildenafil citrate 25 mg three times a day for three months.	Insulin-stimulated AKT Phosphorylation	NCT02129725
Obesity Fatty Liver, Nonalcoholic	2014	Not Applicable	Drug: pitavastatin	Pitavastatin 4 mg daily by mouth for 6 months	Insulin-stimulated Glucose Uptake|Liver Fat|Alanine Aminotransferase|Aspartate Aminotransferase|Hepatic Insulin Sensitivity|Hemoglobin A1c|Quantitative Insulin Sensitivity Check Index	NCT02290106
Diabetes Mellitus Type 2	2015	Phase 2	Drug: Momordica charantia	Two 500 mg capsules of Momordica Charantia twice daily before breakfast and dinner for 90 days	Total and First Phase of Insulin Secretion (Insulinogenic Index and Stumvoll Index) After 90 Days|Insulin Sensitivity (Matsuda Index) After 90 Days	NCT02397447

#### Preclinical studies for IR intervention

In recent years, accumulating preclinical studies on the intervention of IR have been reported, which have important reference significance for the development of new drugs. We present the related studies on IR reported in recent years in Table 3, including animal models, treatment methods and results. Pre-clinical IR intervention mainly includes drug intervention, probiotic therapy and exercise supplement. Drug therapy to improve IR is the main research direction at present. Researchers found that Valdecoxib (VAL) can inhibit inflammation and endoplasmic reticulum (ER) stress through AMPK-regulated HSPB1 pathway, thus improving skeletal muscle IR under hyperlipidemia.^501^ The insulin signal described above has a key role in AD pathogenesis. The researchers found that the mixed nasal administration of GLP-1 receptor agonist and L-form of peneracin can effectively alleviate the cognitive dysfunction of SAMP8 mice.^502^ In addition, studies have found that probiotics can adjust the changes of intestinal flora to reduce inflammation and IR, so probiotics may be an important supplement for the treatment of IR. Natividad et al.^503^ found that HFD-fed mice intervention by Lactobacilllus reuteri strain could effectively alleviate IR. Regular exercise is an alternative intervention measure to maintain the blood sugar level in the normal range and reduce the risk factors. Hsu and colleagues^504^ found that exercise combined with probiotics intervention can have a positive effect on blood sugar and increase insulin sensitivity in mice. The above results show that drug intervention, probiotic supplementation and intensive exercise can improve IR but more clinical data are still needed.

**Table 3:** Selected preclinical studies for insulin resistance

Intervention	Model	Method	Results	Reference
Propranolol and L-D-ISOPROT	Swiss albino mice of high fructose and high fat diet (HFrHFD) model	The mice were injected with propranolol (30 mg/kg/d, i.p.) and low-dose isoproterenol (5 mg/kg/day, i.p.) for 4 weeks after the 13th week of HFrHFD feeding.	Propranolol and l-d- Isopropionic acid can reduce IR of HFrHFD mice by up-regulating β -inhibin 2 signaling activity.	^505^
Sarsasapogenin (ZGY)	High-fat diet (HFD) C57BL/6 J mice; LPS-induced acute adipose tissue inflammation model	ZGY treatment (80 mg/kg/d, ig, lasting for 18 days) can significantly inhibit the acute adipose tissue inflammation of LPS-treated mice. In obese mice fed with high-fat diet, taking ZGY orally (80 mg·kg/d for 6 weeks) can reduce the infiltration of macrophages, improve IRand reduce the inflammation of adipose tissue.	ZGY can improve IR and reduce fat inflammation in HFD mice, which may be related to the inhibition of IKK/NF-κB and JNK inflammatory signaling pathway.	^506^
PPARα/γ dual Agonist: Propane-2-sulfonic acid octadec-9-enyl-amide (N15)	High fat diet and streptozotocin (STZ)-induced diabetic mice	The mice were received single daily oral treatment with N15 (50 or 100 mg/kg, respectively) for 6 weeks.	The anti-IR effect of N15 may be depended on PPARγ pathway.	^507^
Valdecoxib (VAL)	HFD-fed mice	HFD - fed mice were orally administered VAL (5 mg/kg, once every 2 days) for 8 weeks.	VAL can inhibit inflammation and endoplasmic reticulum stress through AMPK-regulated HSPB1 pathway, thus improving skeletal muscle IR under hyperlipidemia.	^501^
D-chiro-Inositol (DCI)	HFD-fed mice	HFD-fed mice were intragastrically administered with 50 mg of DCI/(kg of body weight (bw))/day for 8 weeks.	DCI decreased the hepatic glucose output and the expression levels of PEPCK and G6Pase through PKCε-IRS/PI3K/AKT signaling pathway in insulin-resistant mice.	^508^
Sitagliptin	HFD-fed SD rat	Rats were given Sitagliptin(100 mg/kg/d) by gavage for 8 consecutive days.	Sitagliptin can significantly inhibit lipid accumulation in blood and liver of rats and improve insulin resistance.	^509^
Muscular resistance, hypertrophy and strength training	HFD-fed Swiss mice	Weight-bearing stair climbing training;Muscle resistance exercise; hypertrophy training and strength training.	Muscle resistance training program can reduce weight, obesity index, adipocyte area and low-grade chronic inflammation, and improve insulin resistance.	^510^
Sodium-glucose cotransporter (SGLT) 2 inhibitor: empagliflflozin	HFD induced obese mice.	HFD with 0.003% empagliflflozin (3 mg/kg bodyweight). And HFD with 0.01% empagliflflozin (10 mg/kg bodyweight).	SGLT2 inhibitor empagliflozin enhances fat utilization and browning by M2 or replacing macrophages activation, and reduces obesity-induced inflammation and insulin resistance.	^511^
Heat shock protein (HSP) 70	HFD-fed C57BL/6 mice	Mice were administered intranasally under isoflurane anesthesia, 10 μl (10 and 40 μg) of the appropriate solution was injected into one nostril, and the mice were supine for 1–2 min, three times a week for 26 days.	4 μg HSP70 significantly improved insulin sensitivity, and 10 μg HSP70 showed a trend of improvement.	^512^
Insulin and exenatide	Male Tg2576 mice	Daily treatment of 0.43 ×10^−3^ IU NovoRapid insulin + 0.075 μg exenatide + 5 μg BSA per mouse was used. The treatment was given 6 days a week for 8 months	Compared with the control mice, the expression of insulin receptor cascade related genes in AD-like mice treated with insulin and exenatide was normalized.	^513^
GLP-1 receptor agonists: exendin-4	Senescence-accelerated mouse (SAMP8)	A proper amount of exendin-4 and L-form of peneracin were respectively dissolved in PBS solution containing 0.001% methylcellulose (MC), and an equal amount of peptide drug and osmin solution were gently mixed. The mice were injected with sodium pentobarbital intraperitoneally and then with exendin-4 and L- penetratin intranasally.	After intranasal administration with L-form of peneracin, the distribution of exendin-4 in the whole brain increased significantly. Through intranasal injection of L-form of peneracin, the delivery of exendin-4 and insulin to the brain may contribute to insulin signal transduction in hippocampus.	^502^
Lactobacilllus reuteri strain	HFD-fed mice	The mice were gavaged daily with 10^9^ CFU of L. reuteri CNCM I-5022 for 12 weeks.	Lactobacillus reuteri improved HOMA-IR and glucose clearance and exhibited better insulin sensitivity in HFD-fed mice.	^503^
Sterilized bififidobacteria	HFD-fed mice	Mice were orally administered with bifidobacteria (200 mg·kg^-1^, 400 mg·kg^-1^) daily for 4 weeks.	Oral glucose tolerance and IR test showed that Bifidobacterium sterilization could improve glucose tolerance and reduce insulin resistance.	^514^
Exercise training combined with Bififidobacterium longum OLP-01	Male C57BL/6 J db/db mice	The mice administered orally at a dosage of 1.03 g per kg per day (1.03 × 10^10^ CFU per kg per day) using a stomach tube. The mice were supplemented with strength training.	Exercise and OLP-01 treatment show that they can reduce blood sugar, increase insulin sensitivity, reduce body fat, improve physical activity and protect liver injury, but have no adverse effects.	^504^

Overall, the increased incidence of IR and the key roles of IR plays in many diseases, urgently require a better understanding of IR pathogenesis in addition to how IR interacts with genetics and different environments. A deeper understanding of IR can be achieved with a more systematic approach involving large-scale omics to study the molecular landscape is of major importance in addition to exploring new intervention strategies to prevent abnormal IR syndrome.

## References

[1] Banting FG, Best CH (2007). The internal secretion of the pancreas. 1922. Indian J. Med. Res..

[2] SANGER F, THOMPSON EO (1953). The amino-acid sequence in the glycyl chain of insulin. The identification of lower peptides from partial hydrolysates. Biochem J..

[3] SANGER F, THOMPSON EO (1953). The amino-acid sequence in the glycyl chain of insulin. II. The investigation of peptides from enzymic hydrolysates. Biochem J..

[4] Kung YT, Du YC, Huang WT, Chen CC, Ke LT (1965). Total synthesis of crystalline bovine insulin. Sci. Sin..

[5] Goeddel DV (1979). Expression in Escherichia coli of chemically synthesized genes for human insulin. Proc. Natl. Acad. Sci. USA.

[6] Vecchio I, Tornali C, Bragazzi NL, Martini M (2018). The discovery of insulin: an important milestone in the history of medicine. Front Endocrinol..

[7] Cheatham B, Kahn CR (1995). Insulin action and the insulin signaling network. Endocr. Rev..

[8] Root HF (1929). Insulin resistance and bronze diabetes. N. Engl. J. Med..

[9] Laakso M, Kuusisto J (2014). Insulin resistance and hyperglycaemia in cardiovascular disease development. Nat. Rev. Endocrinol..

[10] Bugianesi E, Moscatiello S, Ciaravella MF, Marchesini G (2010). Insulin resistance in nonalcoholic fatty liver disease. Curr. Pharm. Des..

[11] Saklayen MG (2018). The global epidemic of the metabolic syndrome. Curr. Hypertens. Rep..

[12] Diamanti-Kandarakis E, Dunaif A (2012). Insulin resistance and the polycystic ovary syndrome revisited: an update on mechanisms and implications. Endocr. Rev..

[13] Stenvers DJ, Scheer FA, Schrauwen P, la Fleur SE, Kalsbeek A (2019). Circadian clocks and insulin resistance. Nat. Rev. Endocrinol..

[14] Freeman AM, Pennings N. Insulin Resistance. 2021 Jul 10. In: StatPearls Internet. Treasure Island (FL): StatPearls Publishing. PMID: 29939616. 29939616

[15] American Diabetes Association. (2021). 3. Prevention or delay of type 2 diabetes: standards of medical care in diabetes-2021. Diabetes Care.

[16] American Diabetes Association. (2021). 9. Pharmacologic approaches to glycemic treatment: Standards of Medical Care in Diabetes-2021. Diabetes Care.

[17] Weiss, M. et al. Insulin biosynthesis, secretion, structure, and structure-activity relationships. In: Feingold KR, Anawalt B, Boyce A, et al., eds. Endotext. South Dartmouth (MA): MDText.com, Inc.; (2014). 25905258

[18] Sanger F (1959). Chemistry of insulin. Science.

[19] Katsoyannis PG (1966). Synthesis of insulin. Science.

[20] Lee J, Pilch PF (1994). The insulin receptor: structure, function, and signaling. Am. J. Physiol..

[21] Pessin JE, Saltiel AR (2000). Signaling pathways in insulin action: molecular targets of insulin resistance. J. Clin. Invest.

[22] Haeusler RA, McGraw TE, Accili D (2018). Biochemical and cellular properties of insulin receptor signalling. Nat. Rev. Mol. Cell Biol..

[23] White, M.F. Mechanisms of insulin action. In Atlas of diabetes (pp. 19-38). Springer, Boston, MA (2012).

[24] Newgard CB, Brady MJ, O’Doherty RM, Saltiel AR (2000). Organizing glucose disposal: emerging roles of the glycogen targeting subunits of protein phosphatase-1. Diabetes.

[25] Beurel E, Grieco SF, Jope RS (2015). Glycogen synthase kinase-3 (GSK3): regulation, actions, and diseases. Pharm. Ther..

[26] Dong XC (2008). Inactivation of hepatic Foxo1 by insulin signaling is required for adaptive nutrient homeostasis and endocrine growth regulation. Cell Metab..

[27] Puigserver P (2003). Insulin-regulated hepatic gluconeogenesis through FOXO1–PGC-1α interaction. Nature.

[28] Vander Haar E, Lee SI, Bandhakavi S, Griffin TJ, Kim DH (2007). Insulin signalling to mTOR mediated by the Akt/PKB substrate PRAS40. Nat. Cell Biol..

[29] Garami A (2003). Insulin activation of Rheb, a mediator of mTOR/S6K/4E-BP signaling, is inhibited by TSC1 and 2. Mol. Cell.

[30] Laplante M, Sabatini DM (2010). mTORC1 activates SREBP-1c and uncouples lipogenesis from gluconeogenesis. Proc. Natl. Acad. Sci. USA.

[31] Han Y (2019). Post-translational regulation of lipogenesis via AMPK-dependent phosphorylation of insulin-induced gene. Nat. Commun..

[32] Calejman CM (2020). mTORC2-AKT signaling to ATP-citrate lyase drives brown adipogenesis and de novo lipogenesis. Nat. Commun..

[33] Xia W (2018). Loss of ABHD15 impairs the anti-lipolytic action of insulin by altering PDE3B stability and contributes to insulin resistance. Cell Rep..

[34] James DE, Stöckli J, Birnbaum MJ (2021). The aetiology and molecular landscape of insulin resistance. Nat. Rev. Mol. Cell Biol..

[35] Tam CS (2012). Defining insulin resistance from hyperinsulinemic-euglycemic clamps. Diabetes care.

[36] Samuel VT, Shulman GI (2012). Mechanisms for insulin resistance: common threads and missing links. Cell.

[37] Ye J (2013). Mechanisms of insulin resistance in obesity. Front Med.

[38] Yaribeygi H, Farrokhi FR, Butler AE, Sahebkar A (2019). Insulin resistance: Review of the underlying molecular mechanisms. J. Cell Physiol..

[39] De Meyts P (2008). The insulin receptor: a prototype for dimeric, allosteric membrane receptors?. Trends Biochem Sci..

[40] Caro JF (1987). Insulin receptor kinase in human skeletal muscle from obese subjects with and without noninsulin dependent diabetes. J. Clin. Invest.

[41] Fröjdö S, Vidal H, Pirola L (2009). Alterations of insulin signaling in type 2 diabetes: a review of the current evidence from humans. Biochim Biophys. Acta.

[42] Fisher SJ, Kahn CR (2003). Insulin signaling is required for insulin’s direct and indirect action on hepatic glucose production. J. Clin. Invest.

[43] Michael MD (2000). Loss of insulin signaling in hepatocytes leads to severe insulin resistance and progressive hepatic dysfunction. Mol. Cell.

[44] Davis R, Aguirre V, Uchida T, Yenush L, White MF (2000). The c-Jun NH2-terminal kinase promotes insulin resistance during association with insulin receptor substrate-1 and phosphorylation of Ser307. J. Biol. Chem..

[45] Carvalho-Filho MA (2005). S-nitrosation of the insulin receptor, insulin receptor substrate 1, and protein kinase B/Akt: a novel mechanism of insulin resistance. Diabetes.

[46] Taniguchi CM, Emanuelli B, Kahn CR (2006). Critical nodes in signalling pathways: insights into insulin action. Nat. Rev. Mol. Cell Biol..

[47] Brachmann SM, Ueki K, Engelman JA, Kahn RC, Cantley LC (2005). Phosphoinositide 3-kinase catalytic subunit deletion and regulatory subunit deletion have opposite effects on insulin sensitivity in mice. Mol. Cell Biol..

[48] Cheatham B (1994). Phosphatidylinositol 3-kinase activation is required for insulin stimulation of pp70 S6 kinase, DNA synthesis, and glucose transporter translocation. Mol. Cell Biol..

[49] Czech MP, Corvera S (1999). Signaling mechanisms that regulate glucose transport. J. Biol. Chem..

[50] Luo J (2006). Loss of class IA PI3K signaling in muscle leads to impaired muscle growth, insulin response, and hyperlipidemia. Cell Metab..

[51] Cong LN (1997). Physiological role of Akt in insulin-stimulated translocation of GLUT4 in transfected rat adipose cells. Mol. Endocrinol..

[52] Xia JY (2015). Targeted induction of ceramide degradation leads to improved systemic metabolism and reduced hepatic steatosis. Cell Metab..

[53] Le Marchand-Brustel Y, Grémeaux T, Ballotti R (1985). & Van Obberghen, E. Insulin receptor tyrosine kinase is defective in skeletal muscle of insulin-resistant obese mice. Nature.

[54] Brozinick JT, Roberts BR, Dohm GL (2003). Defective signaling through Akt-2 and-3 but not Akt-1 in insulin-resistant human skeletal muscle: potential role in insulin resistance. Diabetes.

[55] Kruszynska YT (2002). Fatty acid-induced insulin resistance: decreased muscle PI3K activation but unchanged Akt phosphorylation. J. Clin. Endocrinol. Metab..

[56] Choi K, Kim YB (2010). Molecular mechanism of insulin resistance in obesity and type 2 diabetes. Korean J. Intern Med..

[57] Kahn BB (1996). Glucose transport: pivotal step in insulin action. Diabetes.

[58] Dimitriadis G, Mitrou P, Lambadiari V, Maratou E, Raptis SA (2011). Insulin effects in muscle and adipose tissue. Diabetes Res Clin. Pract..

[59] Shepherd PR, Kahn BB (1999). Glucose transporters and insulin action-implications for insulin resistance and diabetes mellitus. N. Engl. J. Med.

[60] Li J, Houseknecht KL, Stenbit AE, Katz EB, Charron MJ (2000). Reduced glucose uptake precedes insulin signaling defects in adipocytes from heterozygous GLUT4 knockout mice. FASEB J..

[61] Klip A (1990). Recruitment of GLUT-4 glucose transporters by insulin in diabetic rat skeletal muscle. Biochem Biophys. Res Commun..

[62] Etgen GJ (1997). Exercise training reverses insulin resistance in muscle by enhanced recruitment of GLUT-4 to the cell surface. Am. J. Physiol..

[63] Ryder JW (2000). Use of a novel impermeable biotinylated photolabeling reagent to assess insulin-and hypoxia-stimulated cell surface GLUT4 content in skeletal muscle from type 2 diabetic patients. Diabetes.

[64] Garvey WT, Maianu L, Zhu JH, Hancock JA, Golichowski AM (1993). Multiple defects in the adipocyte glucose transport system cause cellular insulin resistance in gestational diabetes: heterogeneity in the number and a novel abnormality in subcellular localization of GLUT4 glucose transporters. Diabetes.

[65] Chadt A (2015). Deletion of both Rab-GTPase–activating proteins TBC14KO and TBC1D4 in mice eliminates insulin-and AICAR-stimulated glucose transport. Diabetes.

[66] Chen S, Wasserman DH, MacKintosh C, Sakamoto K (2011). Mice with AS160/TBC1D4-Thr649Ala knockin mutation are glucose intolerant with reduced insulin sensitivity and altered GLUT4 trafficking. Cell Metab..

[67] Tramunt B (2020). Sex differences in metabolic regulation and diabetes susceptibility. Diabetologia.

[68] Greenhill C (2018). Sex differences in insulin resistance. Nat. Rev. Endocrinol..

[69] Qiu J (2018). Estradiol protects proopiomelanocortin neurons against insulin resistance. Endocrinology.

[70] Zidon TM (2020). Effects of ERβ and ERα on OVX-induced changes in adiposity and insulin resistance. J. Endocrinol..

[71] Ikeda K, Horie-Inoue K, Inoue S (2019). Functions of estrogen and estrogen receptor signaling on skeletal muscle. J. Steroid Biochem Mol. Biol..

[72] Gerdts E, Regitz-Zagrosek V (2019). Sex differences in cardiometabolic disorders. Nat. Med.

[73] Chia CW, Egan JM, Ferrucci L (2018). Age-related changes in glucose metabolism, hyperglycemia, and cardiovascular risk. Circ. Res..

[74] Shi H, Kumar SPDS (2012). Sex differences in obesity-related glucose intolerance and insulin resistance. Glucose Tolerance.

[75] Geer EB, Shen W (2009). Gender differences in insulin resistance, body composition, and energy balance. Gend. Med..

[76] Christen T (2018). Sex differences in body fat distribution are related to sex differences in serum leptin and adiponectin. Peptides.

[77] Palmisano BT, Zhu L, Eckel RH, Stafford JM (2018). Sex differences in lipid and lipoprotein metabolism. Mol. Metab..

[78] Kodama K (2013). Ethnic differences in the relationship between insulin sensitivity and insulin response: a systematic review and meta-analysis. Diabetes Care.

[79] Raygor V (2019). Impact of race/ethnicity on insulin resistance and hypertriglyceridaemia. Diab Vasc. Dis. Res.

[80] Sumner AE, Cowie CC (2008). Ethnic differences in the ability of triglyceride levels to identify insulin resistance. Atherosclerosis.

[81] Tan VM (2015). Ethnic differences in insulin sensitivity and beta-cell function among Asian men. Nutr. Diabetes.

[82] Ministry of Health Singapore MOHS. National Health Survey 2010: Available at: http://www.moh.gov.sg/content/dam/moh_web/Publications/Reports/2011/NHS2010%20-%20low%20res.pdf.

[83] Potts J, Simmons D (1994). Sex and ethnic group differences in fat distribution in young United Kingdom South Asians and Europids. J. Clin. Epidemiol..

[84] Ehtisham S, Crabtree N, Clark P, Shaw N, Barrett T (2005). Ethnic differences in insulin resistance and body composition in United Kingdom adolescents. J. Clin. Endocrinol. Metab..

[85] Lear SA, Kohli S, Bondy GP, Tchernof A, Sniderman AD (2009). Ethnic variation in fat and lean body mass and the association with insulin resistance. J. Clin. Endocrinol. Metab..

[86] Mason C (2011). Dietary weight loss and exercise effects on insulin resistance in postmenopausal women. Am. J. Prev. Med.

[87] Mikusova V (2021). Insulin resistance and need for a lifestyle change to eliminate it. Bratisl. Lek. Listy.

[88] orpeleijn E, Saris WH, Blaak EE (2009). Metabolic flexibility in the development of insulin resistance and type 2 diabetes: effects of lifestyle. Obes. Rev..

[89] Shigeta H, Shigeta M, Nakazawa A, Nakamura N, Yoshikawa T (2001). Lifestyle, obesity, and insulin resistance. Diabetes Care.

[90] Oosterman JE, Wopereis S, Kalsbeek A (2020). The circadian clock, shift work, and tissue-specific insulin resistance. Endocrinology.

[91] McAuley KA (2002). Intensive lifestyle changes are necessary to improve insulin sensitivity: a randomized controlled trial. Diabetes care.

[92] Bergman BC (2012). Novel and reversible mechanisms of smoking-induced insulin resistance in humans. Diabetes.

[93] Kan C (2013). A systematic review and meta-analysis of the association between depression and insulin resistance. Diabetes care.

[94] Sung CC, Liao MT, Lu KC, Wu CC (2012). Role of vitamin D in insulin resistance. J. Biomed. Biotechnol..

[95] Ardabili HR, Gargari BP, Farzadi L (2012). Vitamin D supplementation has no effect on insulin resistance assessment in women with polycystic ovary syndrome and vitamin D deficiency. Nutr. Res.

[96] Pasieka AM, Rafacho A (2016). Impact of glucocorticoid excess on glucose tolerance: clinical and preclinical evidence. Metabolites.

[97] Rizza RA, Mandarino LJ, Gerich JE (1982). Cortisol-induced insulin resistance in man: impaired suppression of glucose production and stimulation of glucose utilization due to a postreceptor defect of insulin action. J. Clin. Endocrinol. Metab..

[98] Rizza RA, Mandarino LJ, Gerich JE (1982). Effects of growth hormone on insulin action in man: mechanisms of insulin resistance, impaired suppression of glucose production, and impaired stimulation of glucose utilization. Diabetes.

[99] Barbour L (2007). A Cellular mechanisms for insulin resistance in normal pregnancy and gestational diabetes. Diabetes Care.

[100] Parichatikanond W, Nishimura A, Nishida M, Mangmool S (2018). Prolonged stimulation of β2-adrenergic receptor with β2-agonists impairs insulin actions in H9c2 cells. J. Pharm. Sci..

[101] Walli R (1998). Treatment with protease inhibitors associated with peripheral insulin resistance and impaired oral glucose tolerance in HIV-1-infected patients. AIDS.

[102] Murata H, Hruz PW, Mueckler M (2000). The mechanism of insulin resistance caused by HIV protease inhibitor therapy. J. Biol. Chem..

[103] Teff KL (2013). Antipsychotic-induced insulin resistance and postprandial hormonal dysregulation independent of weight gain or psychiatric disease. Diabetes.

[104] Bittencourt MS, Hajjar LA (2015). Insulin therapy in insulin resistance: could it be part of a lethal pathway?. Atherosclerosis.

[105] Elbein SC, Hasstedt SJ, Wegner K, Kahn SE (1999). Heritability of pancreatic beta-cell function among nondiabetic members of Caucasian familial type 2 diabetic kindreds. J. Clin. Endocrinol. Metab..

[106] Shulman GI (2000). Cellular mechanisms of insulin resistance. J. Clin. Invest.

[107] Knauf C (2005). Brain glucagon-like peptide-1 increases insulin secretion and muscle insulin resistance to favor hepatic glycogen storage. J. Clin. Invest.

[108] Petersen MC, Vatner DF, Shulman GI (2017). Regulation of hepatic glucose metabolism in health and disease. Nat. Rev. Endocrinol..

[109] Matsumoto M, Han S, Kitamura T, Accili D (2006). Dual role of transcription factor FoxO1 in controlling hepatic insulin sensitivity and lipid metabolism. J. Clin. Invest.

[110] Shimomura I (2000). Decreased IRS-2 and increased SREBP-1c lead to mixed insulin resistance and sensitivity in livers of lipodystrophic and ob/ob mice. Mol. Cell.

[111] Petersen KF, Laurent D, Rothman DL, Cline GW, Shulman GI (1998). Mechanism by which glucose and insulin inhibit net hepatic glycogenolysis in humans. J. Clin. Invest.

[112] Henriksen EJ, Dokken BB (2006). Role of glycogen synthase kinase-3 in insulin resistance and type 2 diabetes. Curr. Drug Targets.

[113] Karim S, Adams DH, Lalor PF (2012). Hepatic expression and cellular distribution of the glucose transporter family. World J. Gastroenterol..

[114] Rencurel F (1996). Requirement of glucose metabolism for regulation of glucose transporter type 2 (GLUT2) gene expression in liver. Biochem J..

[115] Thorens BGLUT2 (2015). glucose sensing and glucose homeostasis. Diabetologia.

[116] Eberlé D, Hegarty B, Bossard P, Ferré P, Foufelle F (2004). SREBP transcription factors: master regulators of lipid homeostasis. Biochimie.

[117] Horton JD, Goldstein JL, Brown MS (2002). SREBPs: activators of the complete program of cholesterol and fatty acid synthesis in the liver. J. Clin. Invest.

[118] Ferré P, Foufelle F (2010). Hepatic steatosis: a role for de novo lipogenesis and the transcription factor SREBP-1c. Diabetes Obes. Metab..

[119] Tobe K (2001). Increased expression of the sterol regulatory element-binding protein-1 gene in insulin receptor substrate-2(-/-) mouse liver. J. Biol. Chem..

[120] Dentin R, Girard J, Postic C (2005). Carbohydrate responsive element binding protein (ChREBP) and sterol regulatory element binding protein-1c (SREBP-1c): two key regulators of glucose metabolism and lipid synthesis in liver. Biochimie.

[121] Dentin R (2004). Hepatic glucokinase is required for the synergistic action of ChREBP and SREBP-1c on glycolytic and lipogenic gene expression. J. Biol. Chem..

[122] Herman MA (2012). A novel ChREBP isoform in adipose tissue regulates systemic glucose metabolism. Nature.

[123] Iizuka K, Bruick RK, Liang G, Horton JD, Uyeda K (2004). Deficiency of carbohydrate response element-binding protein (ChREBP) reduces lipogenesis as well as glycolysis. Proc. Natl Acad. Sci. USA.

[124] Jaworski K, Sarkadi-Nagy E, Duncan RE, Ahmadian M, Sul HS (2007). Regulation of triglyceride metabolism. IV. Hormonal regulation of lipolysis in adipose tissue. Am. J. Physiol. Gastrointest. Liver Physiol..

[125] Vaughan M, Berger JE, Steinberg D (1964). Hormone-sensitive lipase and monoglyceride lipase activities in adipose tissue. J. Biol. Chem..

[126] Zmuda-Trzebiatowska E, Oknianska A, Manganiello V, Degerman E (2006). Role of PDE3B in insulin-induced glucose uptake, GLUT-4 translocation and lipogenesis in primary rat adipocytes. Cell Signal.

[127] Choi YH (2006). Alterations in regulation of energy homeostasis in cyclic nucleotide phosphodiesterase 3B–null mice. J. Clin. Invest.

[128] Martinez-Botas J (2000). Absence of perilipin results in leanness and reverses obesity in Lepr db/db mice. Nat. Genet.

[129] Tansey JT (2001). Perilipin ablation results in a lean mouse with aberrant adipocyte lipolysis, enhanced leptin production, and resistance to diet-induced obesity. Proc. Natl. Acad. Sci. USA.

[130] Petersen MC, Shulman GI (2018). Mechanisms of Insulin Action and Insulin Resistance. Physiol. Rev..

[131] Kimball SR, Vary TC, Jefferson LS (1994). Regulation of protein synthesis by insulin. Annu. Rev. Physiol..

[132] Pösö AR, Wert JJ, Mortimore GE (1982). Multifunctional control of amino acids of deprivation-induced proteolysis in liver. Role of leucine. J. Biol. Chem..

[133] Marshall S, Garvey WT, Traxinger RR (1991). New insights into the metabolic regulation of insulin action and insulin resistance: role of glucose and amino acids. FASEB J..

[134] Rudrappa SS (2016). Human skeletal muscle disuse atrophy: effects on muscle protein synthesis, breakdown, and insulin resistance-a qualitative review. Front Physiol..

[135] Medeiros C (2011). Exercise training reduces insulin resistance and upregulates the mTOR/p70S6k pathway in cardiac muscle of diet‐induced obesity rats. J. Cell Physiol..

[136] Antuna-Puente B, Feve B, Fellahi S, Bastard JP (2008). Adipokines: the missing link between insulin resistance and obesity. Diabetes Metab..

[137] Rabe K, Lehrke M, Parhofer KG, Broedl UC (2008). Adipokines and insulin resistance. Mol. Med.

[138] Pessin JE, Kwon H (2013). Adipokines mediate inflammation and insulin resistance. Front Endocrinol. (Lausanne).

[139] Li S, Shin HJ, Ding EL, van Dam RM (2009). Adiponectin levels and risk of type 2 diabetes: a systematic review and meta-analysis. JAMA.

[140] Hotta K (2001). Circulating concentrations of the adipocyte protein adiponectin are decreased in parallel with reduced insulin sensitivity during the progression to type 2 diabetes in rhesus monkeys. Diabetes.

[141] Takahashi M (2008). Chemerin enhances insulin signaling and potentiates insulin-stimulated glucose uptake in 3T3-L1 adipocytes. FEBS Lett..

[142] Yamauchi T (2007). Targeted disruption of AdipoR1 and AdipoR2 causes abrogation of adiponectin binding and metabolic actions. Nat. Med..

[143] Li L (2006). Changes and relations of circulating visfatin, apelin, and resistin levels in normal, impaired glucose tolerance, and type 2 diabetic subjects. Exp. Clin. Endocrinol. Diabetes.

[144] Soriguer F (2009). Apelin levels are increased in morbidly obese subjects with type 2 diabetes mellitus. Obes. Surg..

[145] Yue P (2010). Apelin is necessary for the maintenance of insulin sensitivity. American journal of physiology. Am. J. Physiol. Endocrinol. Metab..

[146] Yue P (2011). Apelin decreases lipolysis via G(q), G(i), and AMPK-dependent mechanisms. Endocrinology.

[147] Segal KR, Landt M, Klein S (1996). Relationship between insulin sensitivity and plasma leptin concentration in lean and obese men. Diabetes.

[148] Amitani M, Asakawa A, Amitani H, Inui A (2013). The role of leptin in the control of insulin-glucose axis. Front Neurosci..

[149] Ceddia RB, Koistinen HA, Zierath JR, Sweeney G (2002). Analysis of paradoxical observations on the association between leptin and insulin resistance. FASEB J..

[150] Covey SD (2006). The pancreatic beta cell is a key site for mediating the effects of leptin on glucose homeostasis. Cell Metab..

[151] Seufert J (2004). Leptin effects on pancreatic beta-cell gene expression and function. Diabetes.

[152] Myers MG, Cowley MA, Münzberg H (2008). Mechanisms of leptin action and leptin resistance. Annu. Rev. Physiol..

[153] Burguera B (2000). Obesity is associated with a decreased leptin transport across the blood-brain barrier in rats. Diabetes.

[154] Gainsford T (1996). Leptin can induce proliferation, differentiation, and functional activation of hemopoietic cells. Proc. Natl. Acad. Sci. USA.

[155] Scherer PE (2006). Adipose tissue: from lipid storage compartment to endocrine organ. Diabetes.

[156] Saponaro C, Gaggini M, Carli F, Gastaldelli A (2015). The subtle balance between lipolysis and lipogenesis: a critical point in metabolic homeostasis. Nutrients.

[157] Frayn KN (2001). Adipose tissue and the insulin resistance syndrome. Proc. Nutr. Soc..

[158] Roden M (1996). Mechanism of free fatty acid-induced insulin resistance in humans. J. Clin. Invest.

[159] Goossens GH (2008). The role of adipose tissue dysfunction in the pathogenesis of obesity-related insulin resistance. Physiol. Behav..

[160] RANDLE PJ, GARLAND PB, HALES CN, NEWSHOLME EA (1963). The glucose fatty-acid cycle. Its role in insulin sensitivity and the metabolic disturbances of diabetes mellitus. Lancet.

[161] Randle PJ, Garland PB, Newsholme EA, Hales CN (1965). The glucose fatty acid cycle in obesity and maturity onset diabetes mellitus. Ann. N. Y Acad. Sci..

[162] Brechtel K (2001). Fast elevation of the intramyocellular lipid content in the presence of circulating free fatty acids and hyperinsulinemia: a dynamic 1H-MRS study. Magn. Reson Med.

[163] Boden G (1997). Role of fatty acids in the pathogenesis of insulin resistance and NIDDM. Diabetes.

[164] Rothman DL (1995). Decreased muscle glucose transport/phosphorylation is an early defect in the pathogenesis of non-insulin-dependent diabetes mellitus. Proc. Natl Acad. Sci. USA.

[165] Cline GW (1999). Impaired glucose transport as a cause of decreased insulin-stimulated muscle glycogen synthesis in type 2 diabetes. N. Engl. J. Med.

[166] Dresner A (1999). Effects of free fatty acids on glucose transport and IRS-1-associated phosphatidylinositol 3-kinase activity. J. Clin. Invest.

[167] Yu C (2002). Mechanism by which fatty acids inhibit insulin activation of insulin receptor substrate-1 (IRS-1)-associated phosphatidylinositol 3-kinase activity in muscle. J. Biol. Chem..

[168] Erion DM, Shulman GI (2010). Diacylglycerol-mediated insulin resistance. Nat. Med.

[169] Kim JK (2004). PKC-theta knockout mice are protected from fat-induced insulin resistance. J. Clin. Invest.

[170] Nagai Y (2009). The role of peroxisome proliferator-activated receptor gamma coactivator-1 beta in the pathogenesis of fructose-induced insulin resistance. Cell Metab..

[171] Yen CL, Monetti M, Burri BJ, Farese RV (2005). The triacylglycerol synthesis enzyme DGAT1 also catalyzes the synthesis of diacylglycerols, waxes, and retinyl esters. J. Lipid Res.

[172] Timmers S, Schrauwen P, de, Vogel J (2008). Muscular diacylglycerol metabolism and insulin resistance. Physiol. Behav..

[173] Liu L (2007). Upregulation of myocellular DGAT1 augments triglyceride synthesis in skeletal muscle and protects against fat-induced insulin resistance. J. Clin. Invest.

[174] Sokolowska E, Blachnio-Zabielska A (2019). The Role of Ceramides in Insulin Resistance. Front Endocrinol. (Lausanne).

[175] Schubert KM, Scheid MP, Duronio V (2000). Ceramide inhibits protein kinase B/Akt by promoting dephosphorylation of serine 473. J. Biol. Chem..

[176] Chaurasia B, Summers SA, Ceramides - (2018). Lipotoxic Inducers of Metabolic Disorders. Trends Endocrinol. Metab..

[177] Stiban J, Tidhar R, Futerman AH (2010). Ceramide synthases: roles in cell physiology and signaling. Adv. Exp. Med Biol..

[178] Stöckli J (2017). Metabolomic analysis of insulin resistance across different mouse strains and diets. J. Biol. Chem..

[179] Blachnio-Zabielska AU, Chacinska M, Vendelbo MH, Zabielski P (2016). The crucial role of C18-Cer in fat-induced skeletal muscle insulin resistance. Cell Physiol. Biochem.

[180] Raichur S (2014). CerS2 haploinsufficiency inhibits β-oxidation and confers susceptibility to diet-induced steatohepatitis and insulin resistance. Cell Metab..

[181] Turpin SM (2014). Obesity-induced CerS6-dependent C16:0 ceramide production promotes weight gain and glucose intolerance. Cell Metab..

[182] Holland WL (2013). An FGF21-adiponectin-ceramide axis controls energy expenditure and insulin action in mice. Cell Metab..

[183] Mente A (2013). Causal relationship between adiponectin and metabolic traits: a Mendelian randomization study in a multiethnic population. PLoS One.

[184] Brown AE, Walker M (2016). Genetics of Insulin Resistance and the Metabolic Syndrome. Curr. Cardiol. Rep..

[185] Chen Z (2020). Functional Screening of Candidate Causal Genes for Insulin Resistance in Human Preadipocytes and Adipocytes. Circ. Res.

[186] Shakhanova A (2020). Association of polymorphism genes ***LPL***, ***ADRB2***, ***AGT*** and ***AGTR1*** with risk of hyperinsulinism and insulin resistance in the Kazakh population. Biomed. Rep..

[187] Henkin L (2003). Genetic epidemiology of insulin resistance and visceral adiposity. The IRAS Family Study design and methods. Ann. Epidemiol..

[188] Parks BW (2015). Genetic architecture of insulin resistance in the mouse. Cell Metab..

[189] Xue A (2018). Genome-wide association analyses identify 143 risk variants and putative regulatory mechanisms for type 2 diabetes. Nat. Commun..

[190] Yousef AA (2018). IRS-1 genetic polymorphism (r.2963 G > A) in type 2 diabetes mellitus patients associated with insulin resistance. Appl Clin. Genet.

[191] Hashemian L, Sarhangi N, Afshari M, Aghaei Meybodi HR, Hasanzad M (2021). The role of the PPARG (Pro12Ala) common genetic variant on type 2 diabetes mellitus risk. J. Diabetes Metab. Disord..

[192] Zeng Q (2021). Association Between Insulin-like Growth Factor-1 rs35767 Polymorphism and Type 2 Diabetes Mellitus Susceptibility: A Meta-Analysis. Front Genet.

[193] Knowles JW (2016). Identification and validation of N-acetyltransferase 2 as an insulin sensitivity gene. J. Clin. Invest.

[194] Richards JB (2009). A genome-wide association study reveals variants in ARL15 that influence adiponectin levels. PLoS Genet.

[195] Eichler EE (2010). Missing heritability and strategies for finding the underlying causes of complex disease. Nat. Rev. Genet.

[196] Flannick J (2019). Exome sequencing of 20,791 cases of type 2 diabetes and 24,440 controls. Nature.

[197] Viñuela A (2020). Genetic variant effects on gene expression in human pancreatic islets and their implications for T2D. Nat. Commun..

[198] Metz S (2022). The Arg82Cys polymorphism of the protein nepmucin implies a role in HDL metabolism. J. Endocr. Soc..

[199] Moltke I (2014). A common Greenlandic TBC1D4 variant confers muscle insulin resistance and type 2 diabetes. Nature.

[200] Huypens P (2016). Epigenetic germline inheritance of diet-induced obesity and insulin resistance. Nat. Genet.

[201] Ling C, Rönn T (2019). Epigenetics in human obesity and type 2 diabetes. Cell Metab..

[202] Ahmed S (2020). The role of DNA methylation in the pathogenesis of type 2 diabetes mellitus. Clin. Epigenetics.

[203] Cierzniak A (2021). DNA methylation in adipocytes from visceral and subcutaneous adipose tissue influences insulin-signaling gene expression in obese individuals. Int J. Obes..

[204] Ling C (2020). Epigenetic regulation of insulin action and secretion-role in the pathogenesis of type 2 diabetes. J. Intern Med.

[205] Zhao J, Goldberg J, Bremner JD, Vaccarino V (2012). Global DNA methylation is associated with insulin resistance: a monozygotic twin study. Diabetes.

[206] Zhou Z, Sun B, Li X, Zhu C (2018). DNA methylation landscapes in the pathogenesis of type 2 diabetes mellitus. DNA methylation Landsc. pathogenesis type 2 diabetes Mellit..

[207] Liu J (2019). An integrative cross-omics analysis of DNA methylation sites of glucose and insulin homeostasis. Nat. Commu.

[208] Gu T (2014). DNA methylation analysis of the insulin-like growth factor-1 (IGF1) gene in Swedish men with normal glucose tolerance and type 2 diabetes. J. Diabetes Metab..

[209] Wittenbecher C (2019). Insulin-like growth factor binding protein 2 (IGFBP-2) and the risk of developing type 2 diabetes. Diabetes.

[210] Haywood NJ, Slater TA, Matthews CJ, Wheatcroft SB (2019). The insulin like growth factor and binding protein family: Novel therapeutic targets in obesity & diabetes. Mol. Metab..

[211] Crujeiras AB (2016). Genome-wide DNA methylation pattern in visceral adipose tissue differentiates insulin-resistant from insulin-sensitive obese subjects. Transl. Res.

[212] Ma X, Kang S (2019). Functional implications of DNA methylation in adipose biology. Diabetes.

[213] Gao H (2018). Long non-coding RNAs associated with metabolic traits in human white adipose tissue. EBioMedicine.

[214] Castellano-Castillo D (2019). Altered adipose tissue DNA methylation status in metabolic syndrome: relationships between global DNA methylation and specific methylation at adipogenic, lipid metabolism and inflammatory candidate genes and metabolic variables. J. Clin. Med..

[215] Samblas M, Milagro FI, Martínez A (2019). DNA methylation markers in obesity, metabolic syndrome, and weight loss. Epigenetics.

[216] Naidoo V, Naidoo M, Ghai M (2018). Cell-and tissue-specific epigenetic changes associated with chronic inflammation in insulin resistance and type 2 diabetes mellitus. Scand. J. Immunol..

[217] Ali M (2022). DNA methylation profile of genes involved in inflammation and autoimmunity correlates with vascular function in morbidly obese adults. Epigenetics.

[218] Panchal SK, Brown L (2020). DNA methylation in adipose tissue and metabolic syndrome. J. Clin. Med..

[219] Petrus P (2018). Adipocyte expression of SLC19A1 links DNA hypermethylation to adipose tissue inflammation and insulin resistance. J. Clin. Endocrinol. Metab..

[220] Ramos-Lopez O (2018). DNA methylation signatures at endoplasmic reticulum stress genes are associated with adiposity and insulin resistance. Mol. Genet Metab..

[221] Zhang Y, Chen Y, Qu H, Wang Y (2019). Methylation of HIF3A promoter CpG islands contributes to insulin resistance in gestational diabetes mellitus. Mol. Genet Genom. Med.

[222] van Dijk S (2018). DNA methylation in blood from neonatal screening cards and the association with BMI and insulin sensitivity in early childhood. Int J. Obes..

[223] Ramos-Lopez O (2018). Endoplasmic reticulum stress epigenetics is related to adiposity, dyslipidemia, and insulin resistance. Adipocyte.

[224] Stillman B (2018). Histone modifications: insights into their influence on gene expression. Cell.

[225] Małodobra-Mazur M, Cierzniak A, Myszczyszyn A, Kaliszewski K, Dobosz T (2021). Histone modifications influence the insulin-signaling genes and are related to insulin resistance in human adipocytes. Int J. Biochem Cell Biol..

[226] Davegårdh C, García-Calzón S, Bacos K, Ling C (2018). DNA methylation in the pathogenesis of type 2 diabetes in humans. Mol. Metab..

[227] David V (2014). Gluco-incretinsregulate beta-cell glucose competence by epigenetic silencing of fxyd3expression. PLoS ONE.

[228] Emamgholipour, S. et al. Acetylation and insulin resistance: a focus on metabolic and mitogenic cascades of insulin signaling. *Crit. Rev. Clin. Lab. Sci*. 1−19 (2020). 10.1080/10408363.2019.1699498 31894999

[229] Emamgholipour S, Ebrahimi R, Bahiraee A, Niazpour F, Meshkani R (2020). Acetylation and insulin resistance: a focus on metabolic and mitogenic cascades of insulin signaling. Crit. Rev. Clin. Lab Sci..

[230] Zhou S, Tang X, Chen HZ (2018). Sirtuins and insulin resistance. Front Endocrinol..

[231] Castellano-Castillo D (2019). Human adipose tissue H3K4me3 histone mark in adipogenic, lipid metabolism and inflammatory genes is positively associated with BMI and HOMA-IR. PloS one.

[232] Maude H, Sanchez-Cabanillas C, Cebola I (2021). Epigenetics of Hepatic Insulin Resistance. Front Endocrinol..

[233] Cech TR, Steitz JA (2014). The noncoding RNA revolution-trashing old rules to forge new ones. Cell.

[234] Formichi C (2021). Non-Coding RNAs: Novel Players in Insulin Resistance and Related Diseases. Int J. Mol. Sci..

[235] Ghafouri-Fard S, Taheri M (2021). The expression profile and role of non-coding RNAs in obesity. Eur. J. Pharm..

[236] O’Brien J, Hayder H, Zayed Y, Peng C (2018). Overview of microRNA biogenesis, mechanisms of actions, and circulation. Front Endocrinol. (Lausanne).

[237] Agbu P, Carthew RW (2021). MicroRNA-mediated regulation of glucose and lipid metabolism. Nat. Rev. Mol. Cell Biol..

[238] Poy MN (2004). A pancreatic islet-specific microRNA regulates insulin secretion. Nature.

[239] Jiménez-Lucena R (2018). A plasma circulating miRNAs profile predicts type 2 diabetes mellitus and prediabetes: from the CORDIOPREV study. Exp. Mol. Med..

[240] Lee CS (2002). Foxa2 controls Pdx1 gene expression in pancreatic β-cells in vivo. Diabetes.

[241] Xu G, Chen J, Jing G, Shalev A (2013). Thioredoxin-interacting protein regulates insulin transcription through microRNA-204. Nat. Med.

[242] Bao L (2015). MicroRNA-185 targets SOCS3 to inhibit beta-cell dysfunction in diabetes. PloS One.

[243] Iacomino G, Siani A (2017). Role of microRNAs in obesity and obesity-related diseases. Genes Nutr..

[244] Suksangrat T, Phannasil P, Jitrapakdee S (2019). miRNA regulation of glucose and lipid metabolism in relation to diabetes and non-alcoholic fatty liver disease. Adv. Exp. Med Biol..

[245] Chen Y (2013). miRNA-93 inhibits GLUT4 and is overexpressed in adipose tissue of polycystic ovary syndrome patients and women with insulin resistance. Diabetes.

[246] Zhou Y (2016). MicroRNA-29a induces insulin resistance by targeting PPARδ in skeletal muscle cells. Int J. Mol. Med.

[247] Wang S (2019). Micro-RNA-27a/b negatively regulates hepatic gluconeogenesis by targeting FOXO1. Am. J. Physiol. Endocrinol. Metab..

[248] Li C (2019). Decreased glycogenolysis by miR-338-3p promotes regional glycogen accumulation within the spinal cord of amyotrophic lateral sclerosis mice. Front Mol. Neurosci..

[249] Zheng H (2021). MicroRNA-185-5p inhibits hepatic gluconeogenesis and reduces fasting blood glucose levels by suppressing G6Pase. Theranostics.

[250] Wagschal A (2015). Genome-wide identification of microRNAs regulating cholesterol and triglyceride homeostasis. Nat. Med..

[251] Rayner KJ (2010). MiR-33 contributes to the regulation of cholesterol homeostasis. Science.

[252] Goedeke L (2015). MicroRNA-148a regulates LDL receptor and ABCA1 expression to control circulating lipoprotein levels. Nat. Med..

[253] Soh J (2013). MicroRNA-30c reduces hyperlipidemia and atherosclerosis in mice by decreasing lipid synthesis and lipoprotein secretion. Nat. Med.

[254] Salerno AG (2020). LDL Receptor Pathway Regulation by miR-224 and miR-520d. Front Cardiovasc Med.

[255] Miranda K (2018). MicroRNA-30 modulates metabolic inflammation by regulating Notch signaling in adipose tissue macrophages. Int J. Obes..

[256] Wang Y (2021). Natural killer cell-derived exosomal miR-1249-3p attenuates insulin resistance and inflammation in mouse models of type 2 diabetes. Signal Transduct. Target Ther..

[257] Quinn JJ, Chang HY (2016). Unique features of long non-coding RNA biogenesis and function. Nat. Rev. Genet.

[258] Squillaro T (2020). Long non-coding RNAs in regulation of adipogenesis and adipose tissue function. Elife.

[259] Zhang TN, Wang W, Yang N, Huang XM, Liu CF (2020). Regulation of glucose and lipid metabolism by long non-coding RNAs: facts and research progress. Front Endocrinol. (Lausanne).

[260] Yang S, Sun J (2018). LncRNA SRA deregulation contributes to the development of atherosclerosis by causing dysfunction of endothelial cells through repressing the expression of adipose triglyceride lipase. Mol. Med Rep..

[261] Liu J, Tang T, Wang GD, Liu B (2019). LncRNA-H19 promotes hepatic lipogenesis by directly regulating miR-130a/PPARγ axis in non-alcoholic fatty liver disease. Biosci. Rep..

[262] Zhang N (2018). Elevated hepatic expression of H19 long noncoding RNA contributes to diabetic hyperglycemia. JCI insight.

[263] Gui W (2020). LncRNAH19 improves insulin resistance in skeletal muscle by regulating heterogeneous nuclear ribonucleoprotein A1. Cell Commun. Signal..

[264] Akerman I (2017). Human pancreatic β cell lncRNAs control cell-specific regulatory networks. Cell Metab..

[265] Xiong L (2020). LncRNA-Malat1 is involved in lipotoxicity-induced ß-cell dysfunction and the therapeutic effect of exendin-4 via Ptbp1. Endocrinology.

[266] Ruan Y (2018). Circulating LncRNAs analysis in patients with type 2 diabetes reveals novel genes influencing glucose metabolism and Islet β-cell function. Cell Physiol. Biochem.

[267] Zhang F (2021). The long non-coding RNA βFaar regulates islet β-cell function and survival during obesity in mice. Nat. Commun..

[268] Li M (2021). A putative long noncoding RNA-encoded micropeptide maintains cellular homeostasis in pancreatic β cells. Mol. Ther. Nucleic Acids.

[269] Li X (2018). The biogenesis, functions, and challenges of circular RNAs. Mol. Cell.

[270] Zaiou M (2020). The emerging role and promise of circular RNAs in obesity and related metabolic disorders. Cells.

[271] Stoll L (2020). A circular RNA generated from an intron of the insulin gene controls insulin secretion. Nat. Commun..

[272] Stoll L (2018). Circular RNAs as novel regulators of β-cell functions in normal and disease conditions. Mol. Metab..

[273] Cai H (2020). Circular RNA HIPK3 contributes to hyperglycemia and insulin homeostasis by sponging miR-192-5p and upregulating transcription factor forkhead box O1. Endocr J..

[274] Sun R, Xue W, Zhao J (2021). Hsa_circ_0054633 mediates apoptosis and insulin secretion in human pancreatic β cells through miR-409-3p/caspase-8 axis. Diabetes Res Clin. Pr..

[275] Chen C (2021). Regulatory roles of circRNAs in adipogenesis and lipid metabolism: emerging insights into lipid-related diseases. FEBS J..

[276] Li A (2018). Identification and characterization of CircRNAs of two pig breeds as a new biomarker in metabolism-related diseases. Cell Physiol. Biochem.

[277] Arcinas C (2019). Adipose circular RNAs exhibit dynamic regulation in obesity and functional role in adipogenesis. Nat. Metab..

[278] Lin X (2021). CircRNF111 Protects Against Insulin Resistance and Lipid Deposition via Regulating miR-143-3p/IGF2R Axis in Metabolic Syndrome. Front Cell Dev. Biol..

[279] Li Q (2019). CircACC1 Regulates Assembly and Activation of AMPK Complex under Metabolic Stress. Cell Metab..

[280] Du R, Wu N, Bai Y, Tang L, Li L (2022). circMAP3K4 regulates insulin resistance in trophoblast cells during gestational diabetes mellitus by modulating the miR-6795-5p/PTPN1 axis. J. Transl. Med.

[281] Nayfach S (2019). New insights from uncultivated genomes of the global human gut microbiome. Nature.

[282] Human Microbiome Project Consortium. (2012). Structure, function and diversity of the healthy human microbiome. Nature.

[283] Derrien M, Alvarez AS, de Vos WM (2019). The gut microbiota in the first decade of life. Trends Microbiol.

[284] Rinninella E (2019). What is the healthy gut microbiota composition? A changing ecosystem across age, environment, diet, and diseases. Microorganisms.

[285] David LA (2014). Diet rapidly and reproducibly alters the human gut microbiome. Nature.

[286] Shi Z (2019). Gut microbiota: An important link between western diet and chronic diseases. Nutrients.

[287] Quiroga R (2020). Exercise training modulates the gut microbiota profile and impairs inflammatory signaling pathways in obese children. Exp. Mol. Med.

[288] Clauss M, Gérard P, Mosca A, Leclerc M (2021). Interplay between exercise and gut microbiome in the context of human health and performance. Front Nutr..

[289] Matenchuk BA, Mandhane PJ, Kozyrskyj AL (2020). Sleep, circadian rhythm, and gut microbiota. Sleep. Med. Rev..

[290] Stenvers DJ (2019). Circadian clocks and insulin resistance. Nat. Rev. Endocrinol..

[291] Ramirez J (2020). Antibiotics as major disruptors of gut microbiota. Front Cell Infect. Microbiol.

[292] Awany D (2019). Host and microbiome genome-wide association studies: current state and challenges. Front Genet.

[293] Cahana I, Iraqi FA (2020). Impact of host genetics on gut microbiome: Take-home lessons from human and mouse studies. Anim. Model Exp. Med.

[294] Lee CJ, Sears CL, Maruthur N (2020). Gut microbiome and its role in obesity and insulin resistance. Ann. N. Y Acad. Sci..

[295] Gérard C (2019). Impact of gut microbiota on host glycemic control. Front Endocrinol..

[296] Del Chierico F (2021). Fecal microbiota signatures of insulin resistance, inflammation, and metabolic syndrome in youth with obesity: a pilot study. Acta Diabetol..

[297] Wu H (2020). The gut microbiota in prediabetes and diabetes: a population-based cross-sectional study. Cell Metab..

[298] Andoh A (2016). Comparison of the gut microbial community between obese and lean peoples using 16 S gene sequencing in a Japanese population. J. Clin. Biochem Nutr..

[299] Agus A, Clément K, Sokol HT (2021). Gut microbiota-derived metabolites as central regulators in metabolic disorders. Gut.

[300] Martin AM, Sun EW, Rogers GB, Keating DJ (2019). The influence of the gut microbiome on host metabolism through the regulation of gut hormone release. Front Physiol..

[301] Scheithauer TP (2020). Gut microbiota as a trigger for metabolic inflammation in obesity and type 2 diabetes. Front Immunol..

[302] de Groot P (2020). Donor metabolic characteristics drive effects of faecal microbiota transplantation on recipient insulin sensitivity, energy expenditure and intestinal transit time. Gut.

[303] Salles BIM, Cioffi D, Ferreira SRG (2020). Probiotics supplementation and insulin resistance: a systematic review. Diabetol. Metab. Syndr..

[304] Johnson JD (2021). On the causal relationships between hyperinsulinaemia, insulin resistance, obesity and dysglycaemia in type 2 diabetes. Diabetologia.

[305] Wilcox G (2005). Insulin and insulin resistance. Clin. Biochem Rev..

[306] Kashyap SR, Defronzo RA (2007). The insulin resistance syndrome: physiological considerations. Diab. Vasc. Dis. Res..

[307] Reaven GM (2005). Insulin resistance, the insulin resistance syndrome, and cardiovascular disease. Panminerva Med..

[308] Gluvic Z (2017). Link between metabolic syndrome and insulin resistance. Curr. Vasc. Pharm..

[309] Ahmed A, Wong RJ, Harrison SA (2015). Nonalcoholic fatty liver disease review: diagnosis, treatment, and outcomes. Clin. Gastroenterol. Hepatol..

[310] Chen Z, Yu R, Xiong Y, Du F, Zhu S (2017). A vicious circle between insulin resistance and inflammation in nonalcoholic fatty liver disease. Lipids Health Dis..

[311] Lonardo A, Ballestri S, Marchesini G, Angulo P, Loria P (2015). Nonalcoholic fatty liver disease: a precursor of the metabolic syndrome. Dig. Liver Dis..

[312] Gross B, Pawlak M, Lefebvre P, Staels B (2017). PPARs in obesity-induced T2DM, dyslipidaemia and NAFLD. Nat. Rev. Endocrinol..

[313] Gao B, Tsukamoto H (2016). Inflammation in alcoholic and nonalcoholic fatty liver disease: friend or foe?. Gastroenterology.

[314] Neuschwander‐Tetri BA (2010). Hepatic lipotoxicity and the pathogenesis of nonalcoholic steatohepatitis: the central role of nontriglyceride fatty acid metabolites. Hepatology.

[315] Petta S (2016). Pathophysiology of non alcoholic fatty liver disease. Int J. Mol. Sci..

[316] Tilg H, Moschen AR (2008). Insulin resistance, inflammation, and non-alcoholic fatty liver disease. Trends Endocrinol. Metab..

[317] Asrih M, Jornayvaz FR (2013). Inflammation as a potential link between nonalcoholic fatty liver disease and insulin resistance. J. Endocrinol..

[318] Cai D (2005). Local and systemic insulin resistance resulting from hepatic activation of IKK-β and NF-κB. Nat. Med.

[319] Khan RS, Bril F, Cusi K, Newsome PN (2019). Modulation of insulin resistance in nonalcoholic fatty liver disease. Hepatology.

[320] Armandi A, Rosso C, Caviglia GP, Bugianesi E (2021). Insulin Resistance across the Spectrum of Nonalcoholic Fatty Liver Disease. Metabolites.

[321] Bugianesi E (2005). Plasma adiponectin in nonalcoholic fatty liver is related to hepatic insulin resistance and hepatic fat content, not to liver disease severity. J. Clin. Endocrinol. Metab..

[322] Moschen AR (2009). Effects of weight loss induced by bariatric surgery on hepatic adipocytokine expression. J. Hepatol..

[323] Kim JY (2007). Obesity-associated improvements in metabolic profile through expansion of adipose tissue. J. Clin. Invest.

[324] Smith GI (2020). Insulin resistance drives hepatic de novo lipogenesis in nonalcoholic fatty liver disease. J. Clin. Invest.

[325] Koo SH, Dutcher AK, Towle HC (2001). Glucose and insulin function through two distinct transcription factors to stimulate expression of lipogenic enzyme genes in liver. J. Biol. Chem..

[326] Samuel VT (2007). Inhibition of protein kinase Cε prevents hepatic insulin resistance in nonalcoholic fatty liver disease. J. Clin. Invest.

[327] Gassaway BM (2018). PKCε contributes to lipid-induced insulin resistance through cross talk with p70S6K and through previously unknown regulators of insulin signaling. Proc. Natl Acad. Sci. USA.

[328] Benhamed F (2012). The lipogenic transcription factor ChREBP dissociates hepatic steatosis from insulin resistance in mice and humans. J. Clin. Invest.

[329] Dentin R (2006). Liver-specific inhibition of ChREBP improves hepatic steatosis and insulin resistance in ob/ob mice. Diabetes.

[330] Legro RS, Castracane VD, Kauffman RP (2004). Detecting insulin resistance in polycystic ovary syndrome: purposes and pitfalls. Obstet. Gynecol. Surv..

[331] Galluzzo A, Amato MC, Giordano C (2008). Insulin resistance and polycystic ovary syndrome. Nutr. Metab. Cardiovasc Dis..

[332] Rojas J (2014). Polycystic ovary syndrome, insulin resistance, and obesity: navigating the pathophysiologic labyrinth. Int J. Reprod. Med.

[333] Wild RA (2002). Long-term health consequences of PCOS. Hum. Reprod. Update.

[334] Diamanti-Kandarakis E, Papavassiliou AG, Kandarakis SA, Chrousos GP (2007). Pathophysiology and types of dyslipidemia in PCOS. Trends Endocrinol. Metab..

[335] Nisenblat V, Norman RJ (2009). Androgens and polycystic ovary syndrome. Curr. Opin. Endocrinol. Diabetes Obes..

[336] Giallauria F (2009). Androgens in polycystic ovary syndrome: the role of exercise and diet[C]. In Seminars in reproductive medicine. Semin Reprod. Med.

[337] Corbould A (2005). Insulin resistance in the skeletal muscle of women with PCOS involves intrinsic and acquired defects in insulin signaling. Am. J. Physiol. Endocrinol. Metab..

[338] Santoro M (2013). Alternative splicing of human insulin receptor gene (INSR) in type I and type II skeletal muscle fibers of patients with myotonic dystrophy type 1 and type 2. Mol. Cell Biochem.

[339] Li T (2017). Role of the PI3K-Akt signaling pathway in the pathogenesis of polycystic ovary syndrome. Reprod. Sci..

[340] Duleba AJ, Dokras A (2012). Is PCOS an inflammatory process?. Fertil. Steril..

[341] Escobar-Morreale HF, Luque-Ramírez M, González F (2011). Circulating inflammatory markers in polycystic ovary syndrome: a systematic review and metaanalysis. Fertil. Steril..

[342] Zhang YF (2006). Elevated serum levels of interleukin-18 are associated with insulin resistance in women with polycystic ovary syndrome. Endocrine.

[343] Gambineri A, Pelusi C, Vicennati V, Pagotto U, Pasquali R (2002). Obesity and the polycystic ovary syndrome. Int J. Obes. Relat. Metab. Disord..

[344] Gonzalez F (2012). Hyperglycemia-induced oxidative stress is independent of excess abdominal adiposity in normal-weight women with polycystic ovary syndrome. Hum. Reprod..

[345] Pusalkar M, Meherji P, Gokral J, Chinnaraj S, Maitra A (2009). CYP11A1 and CYP17 promoter polymorphisms associate with hyperandrogenemia in polycystic ovary syndrome. Fertil. Steril..

[346] Wickenheisser JK (2000). Differential activity of the cytochrome P450 17α-hydroxylase and steroidogenic acute regulatory protein gene promoters in normal and polycystic ovary syndrome theca cells. J. Clin. Endocrinol. Metab..

[347] Diamanti-Kandarakis E, Papavassiliou AG (2006). Molecular mechanisms of insulin resistance in polycystic ovary syndrome. Trends Mol. Med.

[348] “Cardiovascular diseases (CVDs)”. World Health Organization. https://www.who.int/news-room/fact-sheets/detail/cardiovascular-diseases-(cvds).

[349] Pearson-Stuttard J (2017). Reducing US cardiovascular disease burden and disparities through national and targeted dietary policies: a modelling study. PLoS Med.

[350] Virani SS (2021). Heart disease and stroke statistics-2021 update: a report from the American Heart Association. Circulation.

[351] Nabel EG (2003). Cardiovascular disease. N. Engl. J. Med.

[352] National Center for Biotechnology Information (US). Genes and Disease [Internet]. Bethesda (MD): National Center for Biotechnology Information (US); 1998-. The Heart and Blood Vessels. Available from: https://www.ncbi.nlm.nih.gov/books/NBK22227/.

[353] Ormazabal V (2018). Association between insulin resistance and the development of cardiovascular disease. Cardiovasc Diabetol..

[354] Shulman GI (2014). Ectopic fat in insulin resistance, dyslipidemia, and cardiometabolic disease. N. Engl. J. Med.

[355] Janus A, Szahidewicz-Krupska E, Mazur G, Doroszko A (2016). Insulin resistance and endothelial dysfunction constitute a common therapeutic target in cardiometabolic disorders. Mediators Inflamm..

[356] Stančáková A (2009). Changes in insulin sensitivity and insulin release in relation to glycemia and glucose tolerance in 6,414 Finnish men. Diabetes.

[357] Rawshani A (2017). Mortality and cardiovascular disease in type 1 and type 2 diabetes. N. Engl. J. Med..

[358] Arner P (2002). Insulin resistance in type 2 diabetes: role of fatty acids. Diabetes Metab. Res Rev..

[359] Van Gaal LF, Mertens IL, Christophe E (2006). Mechanisms linking obesity with cardiovascular disease. Nature.

[360] Montani JP (2004). Ectopic fat storage in heart, blood vessels and kidneys in the pathogenesis of cardiovascular diseases. Int J. Obes. Relat. Metab. Disord..

[361] Wende AR, Abel ED (2010). Lipotoxicity in the heart. Biochim Biophys. Acta.

[362] Hedayatnia M (2020). Dyslipidemia and cardiovascular disease risk among the MASHAD study population. Lipids Health Dis..

[363] Nicholls, S. & Lundman, P. Practical Approach to Diagnosis & Management of Lipid Disorders. Jones & Bartlett Learning (2009).

[364] Hartge MM, Unger T, Kintscher U (2007). The endothelium and vascular inflammation in diabetes. Diab Vasc. Dis. Res.

[365] Hsueh WA, Quiñones MJ (2003). Role of endothelial dysfunction in insulin resistance. Am. J. Cardiol..

[366] Aroor AR, Mandavia CH, Sowers JR (2012). Insulin resistance and heart failure: molecular mechanisms. Heart Fail Clin..

[367] 2021 Alzheimer’s disease facts and figures. *Alzheimers Dement*. **17**, 327−406 (2021). 10.1002/alz.12328 33756057

[368] Nguyen TT, Ta Q, Nguyen T, Le TT, Vo VG (2020). Role of Insulin Resistance in the Alzheimer’s Disease Progression. Neurochem Res.

[369] De Felice FG, Lourenco MV, Ferreira ST (2014). How does brain insulin resistance develop in Alzheimer’s disease?. Alzheimers Dement.

[370] Fernandez AM, Torres-Alemán I (2012). The many faces of insulin-like peptide signalling in the brain. Nat. Rev. Neurosci..

[371] Zhao WQ, Chen H, Quon MJ, Alkon DL (2004). Insulin and the insulin receptor in experimental models of learning and memory. Eur. J. Pharm..

[372] Dou JT, Chen M, Dufour F, Alkon DL, Zhao WQ (2005). Insulin receptor signaling in long-term memory consolidation following spatial learning. Learn Mem..

[373] Banks WA, Jaspan JB, Huang W, Kastin AJ (1997). Transport of insulin across the blood-brain barrier: saturability at euglycemic doses of insulin. Peptides.

[374] Baura GD (1993). Saturable transport of insulin from plasma into the central nervous system of dogs in vivo. A mechanism for regulated insulin delivery to the brain. J. Clin. Invest.

[375] Banks WA (2004). The source of cerebral insulin. Eur. J. Pharm..

[376] Pardridge WM, Eisenberg J, Yang J (1985). Human blood-brain barrier insulin receptor. J. Neurochem..

[377] Goldstein BJ (2002). Insulin resistance as the core defect in type 2 diabetes mellitus. Am. J. Cardiol..

[378] Mielke JG (2005). A biochemical and functional characterization of diet-induced brain insulin resistance. J. Neurochem.

[379] Arnold SE (2018). Brain insulin resistance in type 2 diabetes and Alzheimer disease: concepts and conundrums. Nat. Rev. Neurol..

[380] Steen E (2005). Impaired insulin and insulin-like growth factor expression and signaling mechanisms in Alzheimer’s disease–is this type 3 diabetes?. J. Alzheimers Dis..

[381] Rivera EJ (2005). Insulin and insulin-like growth factor expression and function deteriorate with progression of Alzheimer’s disease: link to brain reductions in acetylcholine. J. Alzheimers Dis..

[382] Demir S, Nawroth PP, Herzig S (2021). & Ekim Üstünel, B. Emerging Targets in Type 2 Diabetes and Diabetic Complications. Adv. Sci. (Weinh.).

[383] Hur J (2011). The identification of gene expression profiles associated with progression of human diabetic neuropathy. Brain.

[384] Salameh TS, Rhea EM, Banks WA, Hanson AJ (2016). Insulin resistance, dyslipidemia, and apolipoprotein E interactions as mechanisms in cognitive impairment and Alzheimer’s disease. Exp. Biol. Med (Maywood).

[385] Liu CC (2015). Neuronal LRP1 regulates glucose metabolism and insulin signaling in the brain. J. Neurosci..

[386] Su F, Bai F, Zhang Z (2016). Inflammatory cytokines and Alzheimer’s disease: a review from the perspective of genetic polymorphisms. Neurosci. Bull..

[387] Bagyinszky E (2017). Role of inflammatory molecules in the Alzheimer’s disease progression and diagnosis. J. Neurol. Sci..

[388] Erol A (2008). An integrated and unifying hypothesis for the metabolic basis of sporadic Alzheimer’s disease. J. Alzheimers Dis..

[389] Suzanne M (2017). Insulin resistance and neurodegeneration: progress towards the development of new therapeutics for Alzheimer’s disease. Drugs.

[390] Ringheim GE (1998). Enhancement of beta-amyloid precursor protein transcription and expression by the soluble interleukin-6 receptor/interleukin-6 complex. Brain Res Mol. Brain Res.

[391] Bauer J (1991). Interleukin-6 and α-2-macroglobulin indicate an acute-phase state in Alzheimer’s disease cortices. FEBS lett..

[392] Erta M, Quintana A, Hidalgo J (2012). Interleukin-6, a major cytokine in the central nervous system. Int J. Biol. Sci..

[393] Landreth G, Jiang Q, Mandrekar S, Heneka M (2008). PPARγ agonists as therapeutics for the treatment of Alzheimer’s disease. Neurotherapeutics.

[394] Remels AH (2009). PPARgamma inhibits NF-kappaB-dependent transcriptional activation in skeletal muscle. Am. J. Physiol. Endocrinol. Metab..

[395] Miller BW, Willett KC, Desilets AR (2011). Rosiglitazone and pioglitazone for the treatment of Alzheimer’s disease. Ann. Pharmacother..

[396] Webster AC, Nagler EV, Morton RL, Masson P (2017). Chronic Kidney Disease. Lancet.

[397] Centers for Disease Control and Prevention. Chronic kidney disease in the United States, 2019. Atlanta, GA: US Department of Health and Human Services, Centers for Disease Control and Prevention (2019).

[398] Foley RN, Parfrey PS, Sarnak M (1998). J. Clinical epidemiology of cardiovascular disease in chronic renal disease. Am. J. Kidney Dis..

[399] Romagnani P (2017). Chronic kidney disease. Nat. Rev. Dis. Prim..

[400] Mirabelli M (2020). Mediterranean Diet Nutrients to Turn the Tide against Insulin Resistance and Related Diseases. Nutrients.

[401] Expert Panel on Detection, Evaluation, and Treatment of High Blood Cholesterol in Adults. (2001). Executive Summary of The Third Report of The National Cholesterol Education Program (NCEP) Expert Panel on Detection, Evaluation, And Treatment of High Blood Cholesterol In Adults (Adult Treatment Panel III). JAMA.

[402] Kurella M, Lo JC, Chertow GM (2005). Metabolic syndrome and the risk for chronic kidney disease among nondiabetic adults. J. Am. Soc. Nephrol..

[403] Ryu S (2009). Time-dependent association between metabolic syndrome and risk of CKD in Korean men without hypertension or diabetes. Am. J. Kidney Dis..

[404] Ikizler TA (2008). Nutrition, inflammation and chronic kidney disease. Curr. Opin. Nephrol. Hypertens..

[405] Borazan A, Binici DN (2010). Relationship between insulin resistance and inflamation markers in hemodialysis patients. Ren. Fail.

[406] Slee AD (2012). Exploring metabolic dysfunction in chronic kidney disease. Nutr. Metab. (Lond.).

[407] Ottonello L (2004). Leptin as a uremic toxin interferes with neutrophil chemotaxis. J. Am. Soc. Nephrol..

[408] Stenvinkel P (1998). Leptin–a new hormone of definite interest for the nephrologist. Nephrol. Dial. Transpl..

[409] Berg AH, Combs TP, Du X, Brownlee M, Scherer PE (2001). The adipocyte-secreted protein Acrp30 enhances hepatic insulin action. Nat. Med.

[410] Yamauchi T (2001). The fat-derived hormone adiponectin reverses insulin resistance associated with both lipoatrophy and obesity. Nat. Med.

[411] Jia T, Carrero JJ, Lindholm B, Stenvinkel P (2012). The complex role of adiponectin in chronic kidney disease. Biochimie.

[412] Teta D (2015). Insulin resistance as a therapeutic target for chronic kidney disease. J. Ren. Nutr..

[413] Mallamaci F, Tripepi G, Cutrupi S, Malatino LS, Zoccali C (2005). Prognostic value of combined use of biomarkers of inflammation, endothelial dysfunction, and myocardiopathy in patients with ESRD. Kidney Int.

[414] Thomas SS, Zhang L, Mitch WE (2015). Molecular mechanisms of insulin resistance in chronic kidney disease. Kidney Int.

[415] Liao MT (2012). Insulin resistance in patients with chronic kidney disease. J. Biomed. Biotechnol..

[416] Arcidiacono B (2012). Insulin resistance and cancer risk: an overview of the pathogenetic mechanisms. Exp. Diabetes Res.

[417] Tsugane S, Inoue M (2010). Insulin resistance and cancer: epidemiological evidence. Cancer Sci..

[418] Godsland IF (2009). Insulin resistance and hyperinsulinaemia in the development and progression of cancer. Clin. Sci. (Lond.).

[419] Djiogue S (2013). Insulin resistance and cancer: the role of insulin and IGFs. Endocr. Relat. Cancer.

[420] Alvino CL (2011). Understanding the mechanism of insulin and insulin-like growth factor (IGF) receptor activation by IGF-II. PLoS One.

[421] Tzivion G, Dobson M, Ramakrishnan G (2011). FoxO transcription factors; Regulation by AKT and 14-3-3 proteins. Biochim Biophys. Acta.

[422] Hao P (2021). IRS4 promotes the progression of non-small cell lung cancer and confers resistance to EGFR-TKI through the activation of PI3K/Akt and Ras-MAPK pathways. Exp. Cell Res.

[423] Fleming HE (2008). Wnt signaling in the niche enforces hematopoietic stem cell quiescence and is necessary to preserve self-renewal in vivo. Cell Stem Cell.

[424] Ashihara E (2009). beta-catenin small interfering RNA successfully suppressed progression of multiple myeloma in a mouse model. Clin. Cancer Res.

[425] Kaaks R, Lukanova A (2001). Energy balance and cancer: the role of insulin and insulin-like growth factor-I. Proc. Nutr. Soc..

[426] Hursting SD, Berger NA (2010). Energy balance, host-related factors, and cancer progression. J. Clin. Oncol..

[427] Mena S, Ortega A, Estrela JM (2009). Oxidative stress in environmental-induced carcinogenesis. Mutat. Res.

[428] Tiganis T (2011). Reactive oxygen species and insulin resistance: the good, the bad and the ugly. Trends Pharm. Sci..

[429] Roberts CK, Hevener AL, Barnard R (2013). J. Metabolic syndrome and insulin resistance: underlying causes and modification by exercise training. Compr. Physiol..

[430] Kahn BB, Flier JS (2000). Obesity and insulin resistance. J. Clin. Invest.

[431] Nagy C, Einwallner E (2018). Study of In Vivo Glucose Metabolism in High-fat Diet-fed Mice Using Oral Glucose Tolerance Test (OGTT) and Insulin Tolerance Test (ITT). J. Vis. Exp..

[432] Stumvoll M (2000). Use of the oral glucose tolerance test to assess insulin release and insulin sensitivity. Diabetes Care.

[433] Eyth, E., Basit, H. & Smith, C. J. Glucose tolerance test. StatPearls [Internet] (2020). 30422510

[434] Zhang Y (2020). The Optimized Calculation Method for Insulin Dosage in an Insulin Tolerance Test (ITT): A Randomized Parallel Control Study. Front Endocrinol. (Lausanne).

[435] Okita K (2014). Usefulness of the insulin tolerance test in patients with type 2 diabetes receiving insulin therapy. J. Diabetes Investig..

[436] Lee P, Greenfield JR, Ho KK (2009). Factors determining inadequate hypoglycaemia during insulin tolerance testing (ITT) after pituitary surgery. Clin. Endocrinol. (Oxf.).

[437] Binder G, Bosk A, Gass M, Ranke MB, Heidemann PH (2004). Insulin tolerance test causes hypokalaemia and can provoke cardiac arrhythmias. Horm. Res.

[438] Virtue S, Vidal-Puig A (2021). GTTs and ITTs in mice: simple tests, complex answers. Nat. Metab..

[439] Gutch M, Kumar S, Razi SM, Gupta KK, Gupta A (2015). Assessment of insulin sensitivity/ resistance. Indian J. Endocrinol. Metab..

[440] Bonora E (2002). HOMA-estimated insulin resistance is an independent predictor of cardiovascular disease in type 2 diabetic subjects: prospective data from the Verona Diabetes Complications Study. Diabetes Care.

[441] Keskin M, Kurtoglu S, Kendirci M, Atabek ME, Yazici C (2005). Homeostasis model assessment is more reliable than the fasting glucose/insulin ratio and quantitative insulin sensitivity check index for assessing insulin resistance among obese children and adolescents. Pediatrics.

[442] Gayoso-Diz P (2013). Insulin resistance (HOMA-IR) cut-off values and the metabolic syndrome in a general adult population: effect of gender and age: EPIRCE cross-sectional study. BMC Endocr. Disord..

[443] Fernström M, Fernberg U, Hurtig-Wennlöf A (2019). Insulin resistance (HOMA-IR) and body fat (%) are associated to low intake of fruit and vegetables in Swedish, young adults: the cross-sectional lifestyle, biomarkers and atherosclerosis study. BMC Nutr..

[444] Vuguin P, Saenger P, Dimartino-Nardi J (2001). Fasting glucose insulin ratio: a useful measure of insulin resistance in girls with premature adrenarche. J. Clin. Endocrinol. Metab..

[445] Katz A (2000). Quantitative insulin sensitivity check index: a simple, accurate method for assessing insulin sensitivity in humans. J. Clin. Endocrinol. Metab..

[446] Chen H, Sullivan G, Quon MJ (2005). Assessing the predictive accuracy of QUICKI as a surrogate index for insulin sensitivity using a calibration model. Diabetes.

[447] Sumner AE, Finley KB, Genovese DJ, Criqui MH, Boston RC (2005). Fasting triglyceride and the triglyceride-HDL cholesterol ratio are not markers of insulin resistance in African Americans. Arch. Intern Med.

[448] Thaane T, Motala AA, Mckune AJ (2019). Lifestyle modification in the management of insulin resistance states in overweight/obesity: the role of exercise training. J. Endocrinol., Metab. Diabetes South Afr..

[449] Matthaei S, Stumvoll M, Kellerer M, Häring HU (2000). Pathophysiology and pharmacological treatment of insulin resistance. Endocr. Rev..

[450] Boden G, Sargrad K, Homko C, Mozzoli M, Stein TP (2005). Effect of a low-carbohydrate diet on appetite, blood glucose levels, and insulin resistance in obese patients with type 2 diabetes. Ann. Intern Med.

[451] Isharwal S, Misra A, Wasir JS, Nigam P (2009). Diet & insulin resistance: a review & Asian Indian perspective. Indian J. Med. Res.

[452] Snel M (2012). Ectopic fat and insulin resistance: pathophysiology and effect of diet and lifestyle interventions. Int J. Endocrinol..

[453] Hawley JA (2004). Exercise as a therapeutic intervention for the prevention and treatment of insulin resistance. Diabetes Metab. Res. Rev..

[454] Fedewa MV, Gist NH, Evans EM, Dishman RK (2014). Exercise and insulin resistance in youth: a meta-analysis. Pediatrics.

[455] Clamp LD, Hume DJ, Lambert EV, Kroff J (2017). Enhanced insulin sensitivity in successful, long-term weight loss maintainers compared with matched controls with no weight loss history. Nutr. Diabetes.

[456] Van der Aa MP (2016). Long-term treatment with metformin in obese, insulin-resistant adolescents: results of a randomized double-blinded placebo-controlled trial. Nutr. Diabetes.

[457] Velazquez EM, Mendoza S, Hamer T, Sosa F, Glueck CJ (1994). Metformin therapy in polycystic ovary syndrome reduces hyperinsulinemia, insulin resistance, hyperandrogenemia, and systolic blood pressure, while facilitating normal menses and pregnancy. Metabolism.

[458] Giannarelli R, Aragona M, Coppelli A, Del Prato S (2003). Reducing insulin resistance with metformin: the evidence today. Diabetes Metab..

[459] Ruegsegger GN (2019). Exercise and metformin counteract altered mitochondrial function in the insulin-resistant brain. JCI Insight.

[460] Lee JO (2012). Metformin regulates glucose transporter 4 (GLUT4) translocation through AMP-activated protein kinase (AMPK)-mediated Cbl/CAP signaling in 3T3-L1 preadipocyte cells. J. Biol. Chem..

[461] Kim HS (2020). Metformin reduces saturated fatty acid-induced lipid accumulation and inflammatory response by restoration of autophagic flux in endothelial cells. Sci. Rep..

[462] MacDonald PE (2002). The multiple actions of GLP-1 on the process of glucose-stimulated insulin secretion. Diabetes.

[463] Vendrell J (2011). Study of the potential association of adipose tissue GLP-1 receptor with obesity and insulin resistance. Endocrinology.

[464] Oliveira J, Lau E, Carvalho D, Freitas P (2017). Glucagon-like peptide-1 analogues-an efficient therapeutic option for the severe insulin resistance of lipodystrophic syndromes: two case reports. J. Med Case Rep..

[465] Tran KL (2017). Overview of glucagon-like peptide-1 receptor agonists for the treatment of patients with type 2 diabetes. Am. Health Drug Benefits.

[466] Knudsen LB, Lau J (2019). The discovery and development of liraglutide and semaglutide. Front Endocrinol. (Lausanne).

[467] Sharma D, Verma S, Vaidya S, Kalia K, Tiwari V (2018). Recent updates on GLP-1 agonists: Current advancements & challenges. Biomed. Pharmacother..

[468] Hansen BB (2020). Oral semaglutide versus injectable glucagon-like peptide-1 receptor agonists: a cost of control analysis. J. Med. Econ..

[469] Lee YS, Jun HS (2016). Anti-inflammatory effects of GLP-1-based therapies beyond glucose control. Mediators Inflamm..

[470] Deacon CF (2019). Physiology and pharmacology of DPP-4 in glucose homeostasis and the treatment of type 2 diabetes. Front Endocrinol. (Lausanne).

[471] Ghorpade DS (2018). Hepatocyte-secreted DPP4 in obesity promotes adipose inflammation and insulin resistance. Nature.

[472] Trzaskalski NA, Fadzeyeva E, Mulvihill EE (2020). Dipeptidyl peptidase-4 at the interface between inflammation and metabolism. Clin. Med. Insights Endocrinol. Diabetes.

[473] Nargis T, Chakrabarti P (2018). Significance of circulatory DPP4 activity in metabolic diseases. IUBMB life.

[474] Hattori S (2020). Omarigliptin decreases inflammation and insulin resistance in a pleiotropic manner in patients with type 2 diabetes. Diabetol. Metab. Syndr..

[475] Drucker DJ (2007). Dipeptidyl peptidase-4 inhibition and the treatment of type 2 diabetes: preclinical biology and mechanisms of action. Diabetes care.

[476] Richter B, Bandeira‐Echtler E, Bergerhoff K, Lerch C (2008). Dipeptidyl peptidase-4 (DPP-4) inhibitors for type 2 diabetes mellitus. Cochrane Database Syst. Rev..

[477] Wilcox CS (2020). Antihypertensive and renal mechanisms of SGLT2 (sodium-glucose linked transporter 2) inhibitors. Hypertension.

[478] Brown E, Heerspink HJ, Cuthbertson DJ, Wilding JP (2021). SGLT2 inhibitors and GLP-1 receptor agonists: established and emerging indications. Lancet.

[479] Beitelshees AL, Leslie BR, Taylor SI (2019). Sodium-glucose cotransporter 2 inhibitors: A case study in translational research. Diabetes.

[480] McGuire DK (2021). Association of SGLT2 inhibitors with cardiovascular and kidney outcomes in patients with type 2 diabetes: a meta-analysis. JAMA Cardiol..

[481] Jurczak MJ (2011). SGLT2 deletion improves glucose homeostasis and preserves pancreatic β-cell function. Diabetes.

[482] Al Jobori H (2018). Empagliflozin treatment is associated with improved β-cell function in type 2 diabetes mellitus. J. Clin. Endocrinol. Metab..

[483] Sugii S (2009). PPARγ activation in adipocytes is sufficient for systemic insulin sensitization. Proc. Natl Acad. Sci. USA.

[484] Astapova O, Leff T (2012). Adiponectin and PPARγ: cooperative and interdependent actions of two key regulators of metabolism. Vitam. Horm..

[485] Fuentes E, Guzmán-Jofre L, Moore-Carrasco R, Palomo I (2013). Role of PPARs in inflammatory processes associated with metabolic syndrome. Mol. Med. Rep..

[486] Saltiel AR, Olefsky JM (1996). Thiazolidinediones in the treatment of insulin resistance and type II diabetes. Diabetes.

[487] Arner P (2003). The adipocyte in insulin resistance: key molecules and the impact of the thiazolidinediones. Trends Endocrinol. Metab..

[488] Goedeke L, Perry RJ, Shulman GI (2019). Emerging pharmacological targets for the treatment of nonalcoholic fatty liver disease, insulin resistance, and type 2 diabetes. Annu Rev. Pharm. Toxicol..

[489] Lewandowski KC, Dąbrowska K, Brzozowska M, Kawalec J, Lewiński A (2019). Metformin paradoxically worsens insulin resistance in SHORT syndrome. Diabetol. Metab. Syndr..

[490] Lakka TA (2020). A 2 year physical activity and dietary intervention attenuates the increase in insulin resistance in a general population of children: the PANIC study. Diabetologia.

[491] Pramono A, Jocken JW, Blaak EE, van Baak MA (2020). The effect of vitamin D supplementation on insulin sensitivity: a systematic review and meta-analysis. Diabetes care..

[492] Baruch A (2020). Antibody-mediated activation of the FGFR1/Klothoβ complex corrects metabolic dysfunction and alters food preference in obese humans. Proc. Natl Acad. Sci. USA.

[493] Sonoda, J., Chen, M. Z. & Baruch, A. FGF21-receptor agonists: an emerging therapeutic class for obesity-related diseases. *Horm Mol Biol Clin Investig*. **30**, j/hmbci.2017.30. issue-2/hmbci-2017-0002/hmbci-2017-0002.xml (2017). 10.1515/hmbci-2017-0002 28525362

[494] Hua Y, Clark S, Ren J, Sreejayan N (2012). Molecular mechanisms of chromium in alleviating insulin resistance. J. Nutr. Biochem.

[495] Dubey P, Thakur V, Chattopadhyay M (2020). Role of minerals and trace elements in diabetes and insulin resistance. Nutrients.

[496] Goldfine AB (2013). A randomised trial of salsalate for insulin resistance and cardiovascular risk factors in persons with abnormal glucose tolerance. Diabetologia.

[497] Caricilli AM, Saad MJ (2013). The role of gut microbiota on insulin resistance. Nutrients.

[498] Saad MJA, Santos A, Prada PO (2016). Linking gut microbiota and inflammation to obesity and insulin resistance. Physiol. (Bethesda).

[499] Kim YA, Keogh JB, Clifton PM (2018). Probiotics, prebiotics, synbiotics and insulin sensitivity. Nutr. Res. Rev..

[500] Horvath A (2020). Effects of a multispecies synbiotic on glucose metabolism, lipid marker, gut microbiome composition, gut permeability, and quality of life in diabesity: a randomized, double-blind, placebo-controlled pilot study. Eur. J. Nutr..

[501] Kim TJ (2021). Valdecoxib improves lipid-induced skeletal muscle insulin resistance via simultaneous suppression of inflammation and endoplasmic reticulum stress. Biochem Pharm..

[502] Kamei N (2018). Effective nose-to-brain delivery of exendin-4 via coadministration with cell-penetrating peptides for improving progressive cognitive dysfunction. Sci. Rep..

[503] Natividad JM (2018). Impaired Aryl Hydrocarbon Receptor Ligand Production by the Gut Microbiota Is a Key Factor in Metabolic Syndrome. Cell Metab..

[504] Hsu YJ, Wu MF, Lee MC, Huang CC (2021). Exercise training combined with Bifidobacterium longum OLP-01 treatment regulates insulin resistance and physical performance in db/db mice. Food Funct..

[505] Ibrahim WS, Ahmed HMS, Mahmoud AAA, Mahmoud MF, Ibrahim I (2021). Propranolol and low-dose isoproterenol ameliorate insulin resistance, enhance beta-arrestin2 signaling, and reduce cardiac remodeling in high-fructose, high-fat diet-fed mice: Comparative study with metformin. Life Sci..

[506] Yu YY (2021). Sarsasapogenin improves adipose tissue inflammation and ameliorates insulin resistance in high-fat diet-fed C57BL/6 J mice. Acta Pharm. Sin..

[507] Ren T (2018). A novel PPARalpha/gamma agonist, propane-2-sulfonic acid octadec-9-enyl-amide, ameliorates insulin resistance and gluconeogenesis in vivo and vitro. Eur. J. Pharm..

[508] Cheng F (2019). d- chiro-Inositol Ameliorates High Fat Diet-Induced Hepatic Steatosis and Insulin Resistance via PKCepsilon-PI3K/AKT Pathway. J. Agric Food Chem..

[509] Shen T (2018). Sitagliptin reduces insulin resistance and improves rat liver steatosis via the SIRT1/AMPKalpha pathway. Exp. Ther. Med.

[510] Guedes JM (2020). Muscular resistance, hypertrophy and strength training equally reduce adiposity, inflammation and insulin resistance in mice with diet-induced obesity. Einstein (Sao Paulo).

[511] Xu L (2017). SGLT2 inhibition by empagliflozin promotes fat utilization and browning and attenuates inflammation and insulin resistance by polarizing M2 macrophages in diet-induced obese mice. EBioMedicine.

[512] Tytell M (2018). Alfalfa-derived HSP70 administered intranasally improves insulin sensitivity in mice. Cell Stress Chaperones.

[513] Robinson A (2019). Combination of insulin with a GLP1 agonist is associated with better memory and normal expression of insulin receptor pathway genes in a mouse model of Alzheimer’s Disease. J. Mol. Neurosci..

[514] Kikuchi K, Ben Othman M, Sakamoto K (2018). Sterilized bifidobacteria suppressed fat accumulation and blood glucose level. Biochem Biophys. Res. Commun..

